# The Role of Lithium Ions on the Solubility of K_4_ *E*_4_ in Ethylenediamine and the Oxidation of the Zintl Anions [E_4_]^4−^ (*E* = Ge, Sn, Pb) as well as [Ge_9_]^4−^

**DOI:** 10.1002/chem.202500592

**Published:** 2025-04-08

**Authors:** Christian E. Fajman, Dominik M. Dankert, Wilhelm Klein, Thomas F. Fässler

**Affiliations:** ^1^ TUM School of Natural Sciences Department Chemie Technische Universität München Lichtenbergstraße 4 Garching Germany; ^2^ Catalysis Research Center Technische Universität München Ernst‐Otto‐Fischer‐Straße 1 Garching Germany

**Keywords:** ^119^Sn NMR, lithium counterions, oxidation reaction, raman spectroscopy, zintl anions

## Abstract

Zintl phases are excellent precursors for nine atom [*E*_9_]^4−^ clusters, which are readily accessible by dissolution of *A*_4_ *E*_9_ phases (*A* = Na–Rb; *E* = Ge–Pb) in ethylenediamine (*en*). In contrast, the binary alkali‐metal tetrel phases of composition *A*_4_ *E*_4_ are insoluble in *en*. Furthermore, Li^+^ cations are rarely investigated as counterions for tetrel element Zintl clusters. We report here that K_4_ *E*_4_, comprising [*E*_4_]^4−^ polyanions (*E* = Ge, Sn, and Pb), which are insoluble in *en*, readily dissolves in *en* in the presence of lithium ions and the four atomic polyanions [*E*_4_]^4−^ are oxidized to nine‐atom [*E*_9_]^4−^ clusters during dissolution. We isolated crystals of [Li(en)_2.5_]_4_[Ge_9_] and [Li(*en*)_2_]_4_[*E*_9_] (*E* = Sn and Pb) with exclusively Li counterions. Furthermore, the alkali‐metal ion exchange of K_4_Ge_9_ with LiCl in *en* results also in the oxidation of [Ge_9_]^4−^ to [Ge_9_‐Ge_9_]^6−^ dimers which were isolated as partially and fully ion‐exchanged salts such as K_2_[Li(*en*)_2_]_4_[Ge_9_‐Ge_9_] and [Li(*en*)_2_]_6.5_[Ge_9_‐Ge_9_], respectively. NMR spectroscopic investigations of solutions of [Sn_9_]^4−^ that contain variable Li:K ratio reveal contact K^+^/[Sn_9_]^4‐^ ion pairs, while Li^+^ ions form solvent‐separated ion pairs. The role of Li^+^ ions on the solubility of Zintl phases and Li^+^ assisted oxidation of Zintl ions is highlighted.

## Graphical abstract

The solubility of K_4_Ge_4_, K_4_Sn_4_, and K_4_Pb_4_ phases and oxidation of comprised four‐atom clusters in ethylenediamine (*en*) is induced by the addition of LiCl. The ^119^Sn NMR spectra of [Sn_9_]^4−^ with Li^+^ and K^+^ counterions indicate a cation‐dependent change in both the chemical shift and coupling constant, strongly suggesting K^+^/[Sn_9_]^4−^ ion pairing, which is not observed for Li^+^ counterions.

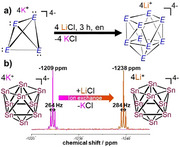

## 1 Introduction

Over the last decades, a series of Zintl phases containing the cluster polyanions [*E*_4_]^4−^ and [*E*_9_]^4−^ (*E* = Si‐Pb) surrounded by an alkali‐metal counterion matrix in *A*_4_ *E*_4_, *A*_4_ *E*_9_, and *A*_12_ *E*_17_ (*A* = Na–Cs) were synthesized and characterized in the solid state.^[^ ^1^, ^2^, ^3^, ^4^, ^5^, ^6^, ^7^ ^]^ Extraction of the solids with polar aprotic solvents led to the characterization of numerous solvates of [*E*_9_]^4−^ thus accounting for the dissolution of the nine‐atom clusters without structural changes.^[^ ^8^, ^9^, ^10^, ^11^, ^12^, ^13^, ^14^, ^15^ ^]^ However, especially germanium clusters with heavier alkali‐metal counterions form larger cluster units by oxidative coupling. Partial oxidation of the nine‐atomic germanium cluster in solution yields extended cluster species with units interconnected by Ge─Ge bonds. Linear oligomers such as dimers [Ge_9_‐Ge_9_]^6−^,^[^ ^16^, ^17^, ^18^, ^19^, ^20^, ^21^, ^22^ ^]^ trimers [Ge_9_ = Ge_9_ = Ge_9_]^6−^,^[^ ^23^, ^24^ ^]^ tetramers [Ge_9_ = Ge_9_ = Ge_9_ = Ge_9_]^8−^,^[^ ^25^, ^26^, ^27^ ^]^ polymeric [‐Ge_9_‐]^2‐[^ ^28^ ^]^ and even more complex cluster arrangements of 45 covalently linked Ge atoms are known.^[^ ^29^ ^]^ Furthermore, a cluster growth through fragmentation is often observed in the presence of organometal compounds resulting in numerous known anions^[^ ^30^, ^31^ ^]^ such as [Co_2_@Ge_16_]^4−^,^[^ ^32^, ^33^ ^]^ [Fe_2_@Ge_16_]^4−^,^[^ ^34^ ^]^ [Pd_2_@Ge_18_]^4‐[^ ^35^ ^]^ and [TM_2_@Ge_17_]^4−^ (TM = Co, Ni).^[^ ^36^ ^]^ Despite these numerous observations on the formation of larger Ge clusters in solution, no clearly defined oxidation agent has been assigned.

The synthetic protocol for the formation of polyanions in solution has not changed much since the introduction of ethylenediamine as a solvent over 50 years ago.^[^ ^37^ ^]^ This is astonishing regarding the numerous reports on Zintl ion formation.^[^ ^30^, ^31^, ^38^, ^39^, ^40^ ^]^ Even though some changes were introduced, such as using 2.2.2.‐crypt,^[^ ^41^ ^]^ 18‐crown‐6^[^ ^13^ ^]^ or by changing the solvent to dimethylformamide^[^ ^19^ ^]^, while the role of the counterions to our knowledge has not been considered yet.

Tetrel element clusters with Li^+^ counterions are comparatively rare, and pure Li_4_ *E*_9_ compounds have not been prepared in solid‐state reactions to date. Intermetallic compounds of composition Li*E* (= Li_4_ *E*_4_) are known for *E* = Si – Pb, however, they comprise extended *E* substructures and do not possess tetrahedral units.^[^ ^42^, ^43^ ^]^ The first indication for “Li_4_ *E*_9_” (*E* = Sn, Pb) in solution was already obtained in 1986 after extracting Li‐*E* alloys with *en* for up to 2 weeks, and the resulting red colored solutions were shown to contain tin and lead clusters via NMR spectroscopy. With Li^+^ and K^+^ counterions, a chemical shift of the NMR signal was observed at −1241 and −1210 ppm for [Sn_9_]^4−^ and at −4188 and −4101 ppm for [Pb_9_]^4−^, respectively.^[^ ^44^ ^]^ Later, Korber et al. directly reduced Sn and Pb with a solution of lithium metal in liquid ammonia to [Li(NH_3_)_4_]_4_[*E*_9_]·NH_3_ (*E* = Sn, Pb) and [Li(NH_3_)_4_]_4_[Sn_4_].^[^ ^45^, ^46^ ^]^ The chemistry of their lighter homologue “Li_4_Ge_9_” has remained completely unexplored and elusive so far, even though Zintl clusters have been investigated for over 100 years. In contrast to the rich chemistry of nine‐atomic clusters, the investigation of tetrahedral clusters in liquid ammonia or *en* consists of a modest amount of compounds, even though these anions were the first polyhedra observed in Na_4_Pb_4_ in the solid state 70 years ago^[^ ^47^ ^]^ and *A*_4_ *E*_4_ phases are known for all element combinations *A*  =  Na‐Rb and *E*  =  Si‐Pb.^[^ ^48^, ^49^, ^50^, ^51^ ^]^ The tetrahedral [*E*_4_]^4−^ cluster exhibits an increased charge‐to‐atom ratio, resulting in a higher reductive potential and lower solubility in comparison to deltahedral [*E*_9_]^4−^ clusters. Therefore most of the chemistry of [*E*_4_]^4−^ is restricted to low temperature liquid ammonia chemistry where some bare^[^ ^46^, ^52^, ^53^, ^54^ ^]^ or transition metal decorated tetrahedral cluster anions such as [(η^3^‐Ge_4_)(ZnEt)_2_]^2−^,^[^ ^55^ ^]^ [(MesCu)_2_(η^3^‐Ge_4_)]^4−^,^[^ ^56^ ^]^ [(NHC*^t^* ^Bu^Au)_6_(η^2^‐Si_4_)]^2+^,^[^ ^57^ ^]^ [(η^2^‐Sn_4_)Zn(η^3^‐Sn_4_)]^6−^,^[^ ^58^ ^]^ and [(η^2^‐Sn_4_)Au(η^2^‐Sn_4_)]^7−[^ ^59^ ^]^ were isolated and structurally characterized. Sun et al. reported that from *en/dmf* solvent mixture of K_12_Ge_17_, known to consist of [Ge_4_]^4−^ and [Ge_9_]^4−^ polyanions, two isostructural cluster aggregates [(η^2^:η^2^:η^2^‐*TM*)_6_(Ge_4_)_4_]^4−^ (*TM* = Zn and Cd) were isolated.^[^ ^60^ ^]^

Whereas the formation of [*E*_9_]^4−^ clusters represents a rare example in which the starting salt is well defined, while, in comparison, the formation of most Zintl ions comprising triel and pentel elements in solution starts from precursors that do not show the same entities as the precursor solids.^[^ ^38^, ^61^ ^]^ Moreover, several larger cluster units are frequently isolated from the same reaction and their formation is justified by oxidation reactions including also cluster fragmentation.^[^ ^30^, ^62^, ^63^ ^]^ Several reports hint at the involvement of *en* in these reactions.^[^ ^64^, ^65^, ^66^ ^]^ Recently, the role of NH_3_ in the oxidation and protonation of *Zintl* ions in liquid ammonia was reported by Korber et al.^[^ ^67^, ^68^, ^69^, ^70^ ^]^

Alkali‐metal counterions are usually regarded as spectator ions in those solutions and are expected to be chemically inert. However, the reported influence of the cations on the ^119^Sn and ^207^Pb NMR chemical shift of *A*_4_ *E*_9_ solutions (*A* = Li to Cs, *E* = Sn, Pb)^[^ ^44^ ^]^ can be interpreted that larger cations diminish the charge density at the cluster by stronger ion pairing with increased alkali‐metal radius and therefore also increase the stability of the anions. Therefore, we investigated whether the lightest alkali‐metal Li increases the reactivity of Zintl anions. In this work, we followed the idea that the alkali‐metal ions play an important role in the chemistry of Zintl ions. Since Li^+^ ions have the highest charge density of all alkali‐metal ions, we present a systematic study on the influence of Li^+^ ions in *en* solutions.

## 2 Results and Discussion

### 2.1 Synthesis of Nine‐Atomic Clusters from *A*_4_ *E*_4_ (*E* = Ge, Sn, Pb)

The new compounds [Li(en)_2.5_]_4_[Ge_9_] (**1**) and [Li(*en*)_2_]_4_[*E*_9_] with *E* = Sn (**2**) or Pb (**3**) as well as [Li_6_(*en*)_13_[Ge_9_‐Ge_9_] (**4**) and K_2_[Li(*en*)_2_]_4_[Ge_9_‐Ge_9_] (**5**), were obtained from reactions of binary Zintl phases K_4_ *E*_4_ (*E* = Ge, Sn, or Pb) or K_4_Ge_9_ in *en*, both in presence of LiCl. For the reaction, two main aspects have to be considered, the exchange of the cation and the oxidation of the Zintl anions. Prior to the syntheses of compounds **1**–**3**, the solubility of the binary solids, that exclusively contain [*E*_4_]^4−^ clusters, in *en* was found to be very low, as indicated by the formation of light orange or even almost colorless supernatant solutions. Upon the addition of LiCl, the color of the solution changes within seconds to the typical deep red color. In the presence of NaCl, the reaction does not proceed. This suggests that the presence of Li^+^, rather than Cl^−^, accelerates the dissolution of the respective K_4_ *E*_4_ phase. In addition, we found that the products **1** to **3** that were isolated from the solutions exclusively contain Li and never K as counter ions. Driving force for this reaction is the high solubility of LiCl in *en* by the solvation of the small Li^+^ ions and the subsequent removal of K^+^ cations from equilibrium by precipitating poorly soluble KCl.^[^ ^71^ ^]^ This is further supported by EDX analysis on the single crystals of compound **1** to **3**, showing the absence of K in the single crystals (see Figure S13 and Table S3).

All products isolated by dissolution of K_4_ *E*_4_ in the presence of LiCl lead to products that contain nine‐atom clusters according to the oxidation reaction 9 E_4_^4−^ —→ 4 E_9_^4−^ + 20 e^−^. A reaction path of this formal oxidation remains unclear so far. In order to identify the formation of [*E*_9_]^4−^ from [*E*_4_]^4−^, the purity of the starting materials K_4_ *E*_4_, *en*, and LiCl were carefully analyzed (see Supporting Information). LiCl and *en* were shown to be anhydrous (Figures S14 and S17), excluding water as the potential oxidizer. The presence of [*E*_9_]^4−^ in the starting material was excluded by Raman spectroscopy and X‐ray diffractograms of K_4_Ge_4_ (Figures S15 and S16). Similarly, no telltale reaction product besides precipitated KCl was detected in the residual solid (Figure S18). However, during the reaction, the weak evolution of a gas observed only in the presence of LiCl hints at the abstraction of H^+^ from *en*, followed by its reduction to H_2_. The observation is supported by the ^1^H NMR spectrum of the reaction solutions, showing a broadening of NH_2_ signal, upon addition of the reactants to an *en* solution. This peak broadening indicates a fast‐fluxional behavior such as a proton exchange reaction (Figure S11 and Table S2), which cannot be resolved on the NMR time scale. A fast proton transfer between deprotonated *en*‐H and the solvent *en* leads to the observed averaged and broad peak for the NH/NH_2_ groups of the solvent. Furthermore, the peak of the NH_2_ group is shifted upfield as a consequence of the shielding of the anionic NH^−^ group, giving a further hint toward the deprotonation of the solvent molecules. Therefore, we propose as a reaction path an activation, that is, an enhanced acidity of *en* by tightly coordinating to Li^+^ cations. The protonation of the [*E*_4_]^4−^ clusters by an *en* molecule of the [Li(*en*)_n_]^+^ complex as the oxidizing agent is proposed as the first reaction step. A possible intermediate of protonated cluster has previously been observed in crystals containing [(μ_2_‐H)(η^2^‐Ge_4_)ZnPh_2_]^3−^ or the bi‐protonated [(μ_2_‐H)_2_(η^2^‐Ge_4_)]^2−^ as well as the protonated [μ‐HSi_4_]^3−^ tetrasilicide identified by NMR spectroscopy in liquid ammonia.^[^ ^68^, ^72^, ^73^ ^]^ In the present case, the reaction in *en* continues with a more comprehensive rearrangement of cluster atoms to the formation of [*E*_9_]^4−^ clusters as a main product. Our proposed reaction path is supported by previous studies about the slow oxidation of [Sn_4_]^4−^ to [Sn_9_]^4−^ clusters by liquid ammonia as a proton source.^[^ ^69^, ^70^ ^]^ In the present case, the reaction runs notably faster as a consequence of the presence of Li^+^ ions and the significantly higher reaction temperature in comparison to the low‐temperature reaction in liquid ammonia. A similar cluster conversion was found after the syntheses of compounds **4** and **5**, starting from [Ge_9_]^4−^ clusters. The principal mechanism, including activated *en* as proton source should be the same as shown above, and also in this case crystallized compounds of nine‐atomic clusters have been found in mono‐ and di‐protonated forms,^[^ ^74^, ^75^ ^]^ but might be intermediate products in this reaction leading to connected pairs of [*E*_9_] clusters. Although the exact mechanism has to be proven, the new LiCl‐assisted method provides a simple and convenient way to prepare the tetrel Zintl cluster compounds with Li^+^ as counterion.

### 2.2 Crystal Structures Determination and Raman Spectroscopic Characterization of the Products

**Figure 1:**
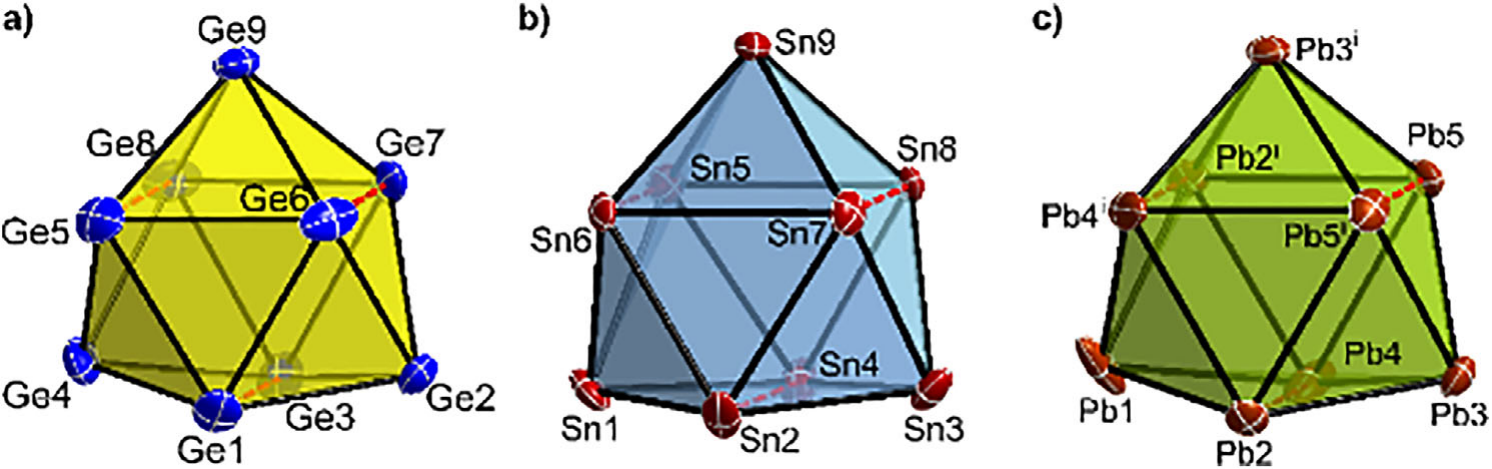
Cluster anions of a) compound 1, b) 2, and c) 3. Elongated bonds representing the heights of the best trigonal prism are indicated by red colored dashed lines. All ellipsoids are shown at a 50% probability level.

After filtration and layering the deeply colored solutions with toluene for crystallization, crystals of compounds **1**, **2**, and **3** were obtained. Despite their similar structural features, the compounds crystallize with slightly different crystal solvent *en* content, different ion packing, and slightly different cluster shapes. In the Ge compound **1**, the anion is disordered and exists in two different orientations with an occupation ratio of 90.08(8)% to 9.92(8)%, and both individuals are slightly more distorted from the fourfold symmetry and, thus, are close to *C*_2_ *_v_* symmetry. The [Sn_9_]^4−^ cluster anion in **2** can be best described as a slightly distorted *C*_4_ *_v_* symmetric monocapped tetragonal antiprism. Finally, half of the [Pb_9_]^4−^ anion (**3**) is generated by a twofold axis of corresponding Pb atoms and the shape of the whole cluster is thus affected by the symmetry, resulting in an almost *D*_3_ *_h_*  symmetric tricapped trigonal prism (Figure 1). The symmetry of the cluster anions **1** to **3** are in good agreement with previously reported bond lengths and angles of the heavier alkali‐metal cation salts (for detailed information about bond lengths, prism heights, etc., see Supporting Information).

**Figure 2:**
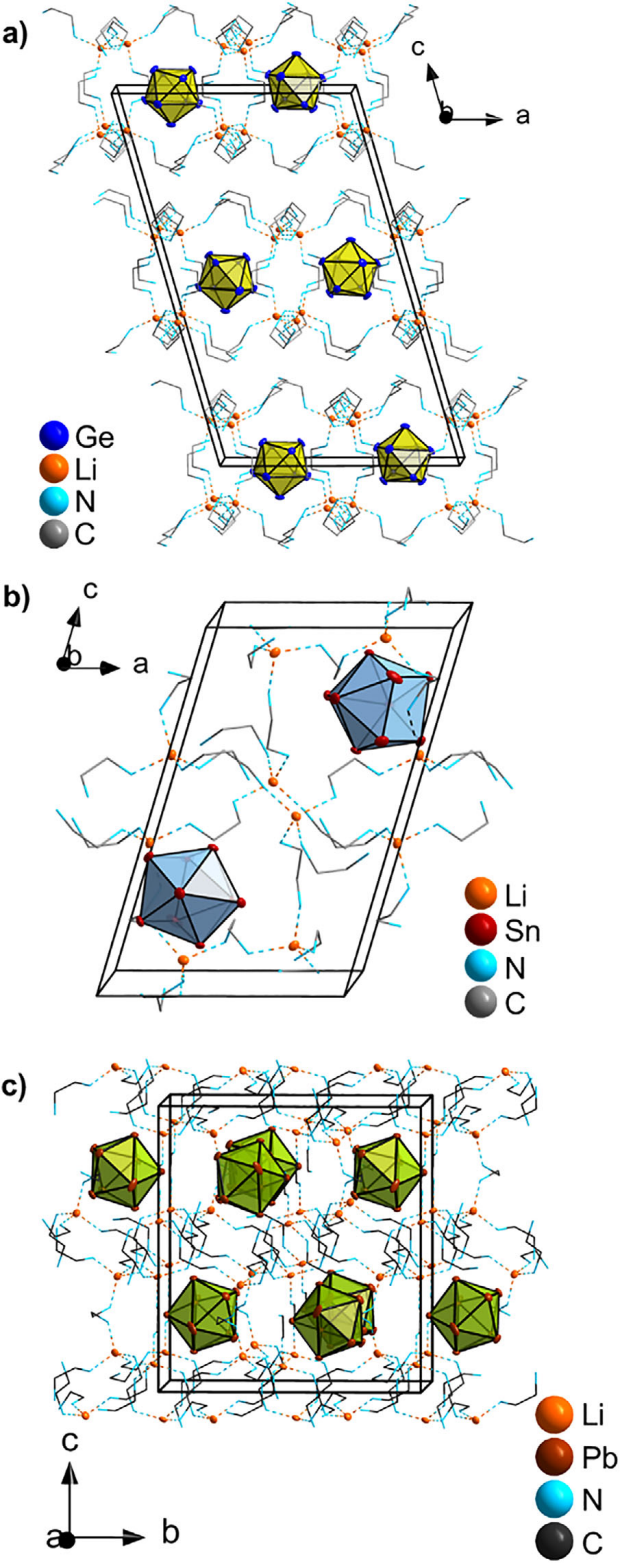
Representation of the extended unit cell of the structures a) **1**, b) **2**, and c) **3**. Ge, Sn, Pb, and Li atoms are shown as blue, red, brown, and orange spheres, respectively. The solvent molecules, including N and C atoms are shown in wire and stick mode. Hydrogen atoms have been omitted for clarity.

In all three compounds, the Li^+^ cations are coordinated by four N atoms of four different *en* molecules in roughly tetrahedral manner. Their observed structural features, such as Li─N bond distances and angles are in good agreement with previously determined values in Li(*en*)_2_ *X* (*X*  =  Cl and Br).^[^ ^76^ ^]^ Furthermore, *en* is also found as a bridging ligand in numerous other structures containing Li(*en*)_4_^+^ complexes.^[^ ^77^, ^78^, ^79^ ^]^ The Li^+^ cation is not bound to a tetrel atom of the cluster anion, since the shortest Li‐*E* distances (*E* = Ge, Sn, Pb) in compound **1** to **3** are 4.1291(1), 4.3273(2) and 4.4198(1) Å, respectively. The coordination of *en* molecules to Li^+^ cations leads to the formation of chains (**1**) and extended 3D arrangements (**2**, **3**). Compound **1** comprises zigzag chains of alternating Li^+^ ions and *en* molecules along the *b‐axis*. The chains are extended orthogonal to the *ab* plane by joining Li^+^ ions through *en* molecules, forming 20‐membered rings. The remaining coordination spheres of the Li^+^ ions are each capped by one *en* molecule. Herein, the [Li_4_(*en*)_10_]^4+^ chains alternate with the [Ge_9_]^4−^ clusters along the *a*‐axis, while the layers of [Li_4_(*en*)_10_][Ge_9_] are not connected along the *c*‐axis (see Figure S19). Different from **1**, in compounds **2** and **3** no “dangling” *en* molecules exist, so all N atoms coordinate to one Li^+^ ion. All Li^+^ cations are bridged by four different *en* molecules, finally forming a three‐dimensional coordination polymer of solvent and counterions around the fourfold negatively charged clusters see Figure 2. In all three structures *en* molecules bridge different cations, thus, there is no chelating effect present.

**Figure 3:**
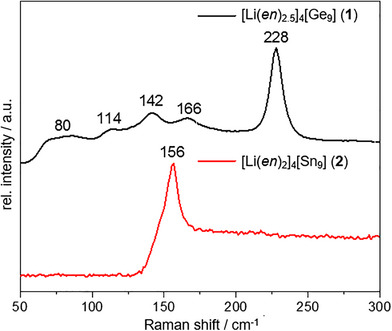
Raman spectra of compound **1** (black) and **2** (red) with the Zintl ions [*E*_9_]^4−^. Characteristic modes are labeled with their corresponding Raman shifts. The spectrum in the range 50– 800 cm^−1^ is shown in the Supporting Information.

According to previous studies, the Raman spectra of [*E*_9_]^4−^ cluster anions show a characteristic, very intense breathing mode at around 222 and 146 cm^−1^ for [Ge_9_]^4−^ and [Sn_9_]^4−^, respectively.^[^ ^7^ ^]^ In order to compare the vibrational behavior of **1** and **2** with their potassium counter parts, single crystals were used for the Raman measurements (Figure 3).

The Raman spectrum of **1** shows one strong mode at 228 cm^−1^ and four weaker modes between 80 and 166 cm^−1^. The location of the modes found in **1** at 80, 114, 142, 166, and 228 cm^−1^ slightly deviate from those reported for solid K_4_Ge_9_ 104, 125, 147, 164, 186, 220, 241 cm^−1^.^[^ ^7^ ^]^ While the frequencies and intensities of the modes at 142, 166, and 228 cm^−1^ match, the modes at 80 and 114 cm^−1^ are absent in K_4_Ge_9_. The additional mode at 80 cm^−1^ was observed in Rb_4_Ge_9_ and Cs_4_Ge_9_ at 81 and 76 cm^−1^, respectively. A so far not observed weak mode at 114 cm^−1^ is also present in the Raman spectra, which cannot be assigned to known vibrations of [Ge_9_]^4−^. For the Raman spectrum of **2** multiple experiments of the highly sensitive compounds were performed, but only spectra with poor intensities, meaning a low signal to background ratio, were recorded. Nevertheless, the mode found in **2** can be approximately assigned to the mode of K_4_Sn_9_, (exp.: 156 cm^−1^ lit.: 99, 146 cm^−1^).^[^ ^7^ ^]^ In summary the main features of the strongest modes are in good agreement with the pure solids. Differences are due to the different local environments of the clusters in pure solids and solvate crystals. The absence of strong additional modes and their good agreement with modes reported in literature are good evidence, that **1** and **2** do contain exclusively [*E*_9_]^4−^ clusters.

### 2.3 Crystal Structures Determination of Ion Exchanged [Ge_9_‐Ge_9_]^6−^ Dimers

**Figure 4:**
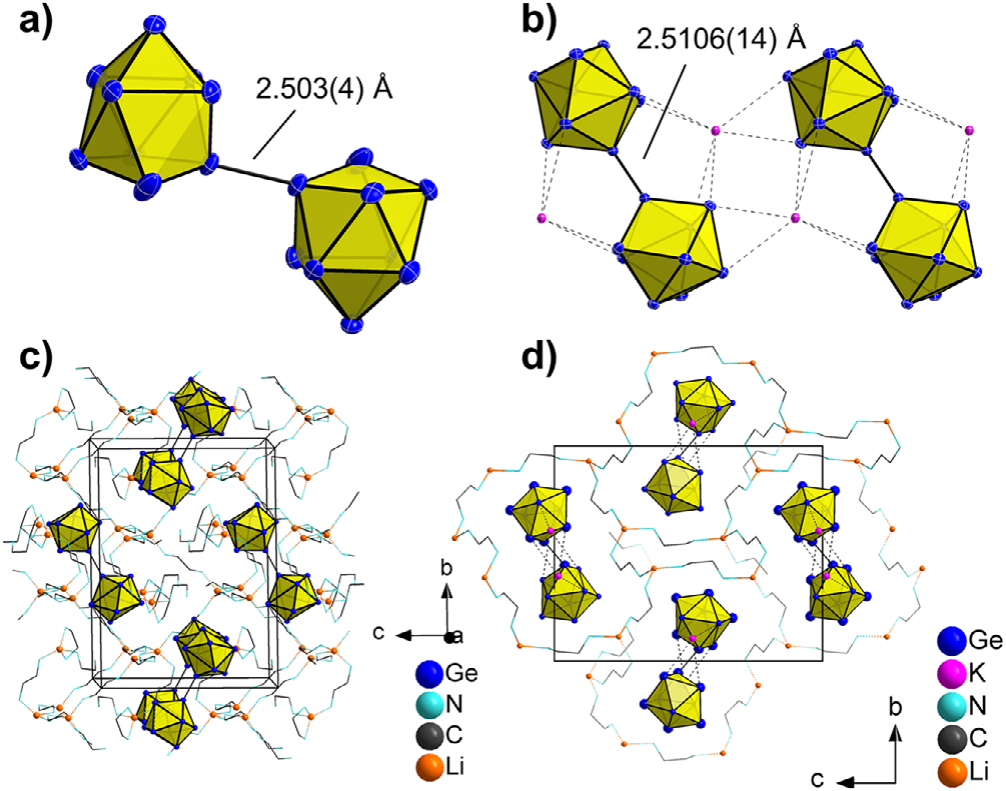
Molecular structure of [Ge_9_]_2_ anions in compounds 4 (a) and 5 (b), the latter together with coordinating K^+^ ions. Unit cells of 4 (c) and of 5 (d), views along the crystallographic *a*‐axis. Ge, Li, and K atoms are shown as blue, orange, and pink spheres, respectively. *En* molecules including N (blue) and C atoms (gray) are shown in wire and stick mode. Hydrogen atoms have been omitted for clarity in (c,d). All ellipsoids are shown at a 50% probability level.

We also investigated the influence of adding LiCl on the dissolution of K_4_ *E*_9_ and further examined the impact of the amount of LiCl added. The addition of four and two equivalents of LiCl to K_4_Ge_9_ in *en* leads to the formation of crystals of two compounds, comprising cluster dimers [Ge_9_‐Ge_9_]^6−^ that crystallize with six and four Li^+^ ions in **4** and **5**, respectively. While the dimer in compound **4** has exclusively six Li^+^ counterions, the anionic dimer **5** is partially ion exchanged, and two K^+^ ions and four Li^+^ ions per cluster are present. Both anions in compounds **4** and **5** consist of one crystallographically independent [Ge_9_] cluster, which is duplicated by an inversion center to give the dimeric anion [Ge_9_‐Ge_9_]^6−^. Each structure of the nine‐atomic building unit of the cluster dimers is close to *C*_4_ *_v_* symmetry and forms a slightly distorted monocapped tetragonal antiprism. The structure of the dimer corresponds to the typical shape and bond lengths of dimeric anions [Ge_9_‐Ge_9_]^6‐^ with heavier alkali‐metal cations (see Table S6).^[^ ^16^, ^17^, ^18^, ^19^, ^20^, ^21^, ^22^ ^]^ In both compounds (**4** and **5**), the anions are interconnected by a covalent Ge‐Ge single bond with a distance of 2.503(4) and 2.5106(14) Å, respectively (Figure 4a,b). In **5,** with two types of cations, interestingly the heavier K^+^ cations coordinate to the Ge atoms of the [Ge_9_‐Ge_9_]^6−^ dimer (Figure 4b), forming one‐dimensional chains of alternating cations and cluster dimers 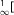 Ge_9_‐Ge_9_K_2_]^4−^ along the crystallographic *a*‐axis (Figure 4d).

Both compounds show complex coordination patterns of *en* molecules linking Li^+^ cations, which form a 3D network of [Li_6_(en)_13_]^6+^ and [Li_4_(en)_8_]^4+^ aggregates (Figure 4c,d). The latter one represents another close network where all Li^+^ ions as well as all N atoms are connected. The anionic clusters are embedded in those matrices of solvent and Li^+^ cations.

### 2.4 NMR Spectroscopic Investigations

In order to study the influence of the cations on the stability and reactivity of the polyanions, solutions of single crystals of compound **1** to **5** were investigated by NMR spectroscopy (^7^Li and ^119^Sn or ^207^Pb NMR). The ^7^Li NMR spectra of **1**–**5** (Figures S1, S2, S4, S6, and S7) show each a singlet with a chemical shift approximately at 1.7 ppm, which is in the typical region of solvated Li^+^ cations. Consequently, no strong ionic interactions between Li^+^ ions and the atoms of the cluster polyanion are expected. This is in good agreement with the [Li(*en*)_4_]^+^ complexes found in the crystal structures, which can be interpreted as fully solvated Li^+^ ions. Further, the ^119^Sn and ^207^Pb NMR spectra of **2** and **3** show shifts at −1239 ppm and −4192 ppm that correspond to [*E*_9_]^4−^ clusters, respectively (Figures S3 and S5). In comparison to the chemical shifts of clusters with corresponding K^+^ salts, the signals of the clusters are significantly high field shifted by 31 and 87 ppm, respectively, which is congruent with the reported values (Figures S8 and S9).^[^ ^44^ ^]^ Additionally, the observed ratios of the central to satellite peak heights (^119^Sn–^117^Sn) of 1:0.31:0.04 for **2** matches the theoretical intensity of a nine‐atomic tin cluster of 1:0.311:0.044. A ^119^Sn–^117^Sn coupling constant of 284 Hz is found for anion **2**, which is slightly higher than the previously reported coupling constant of 273 Hz for the Li^+^ salt. Additionally, an increase of the coupling constant in comparison to the coupling constant reported for the K^+^ salt (263 Hz) is found.

**Figure 5:**
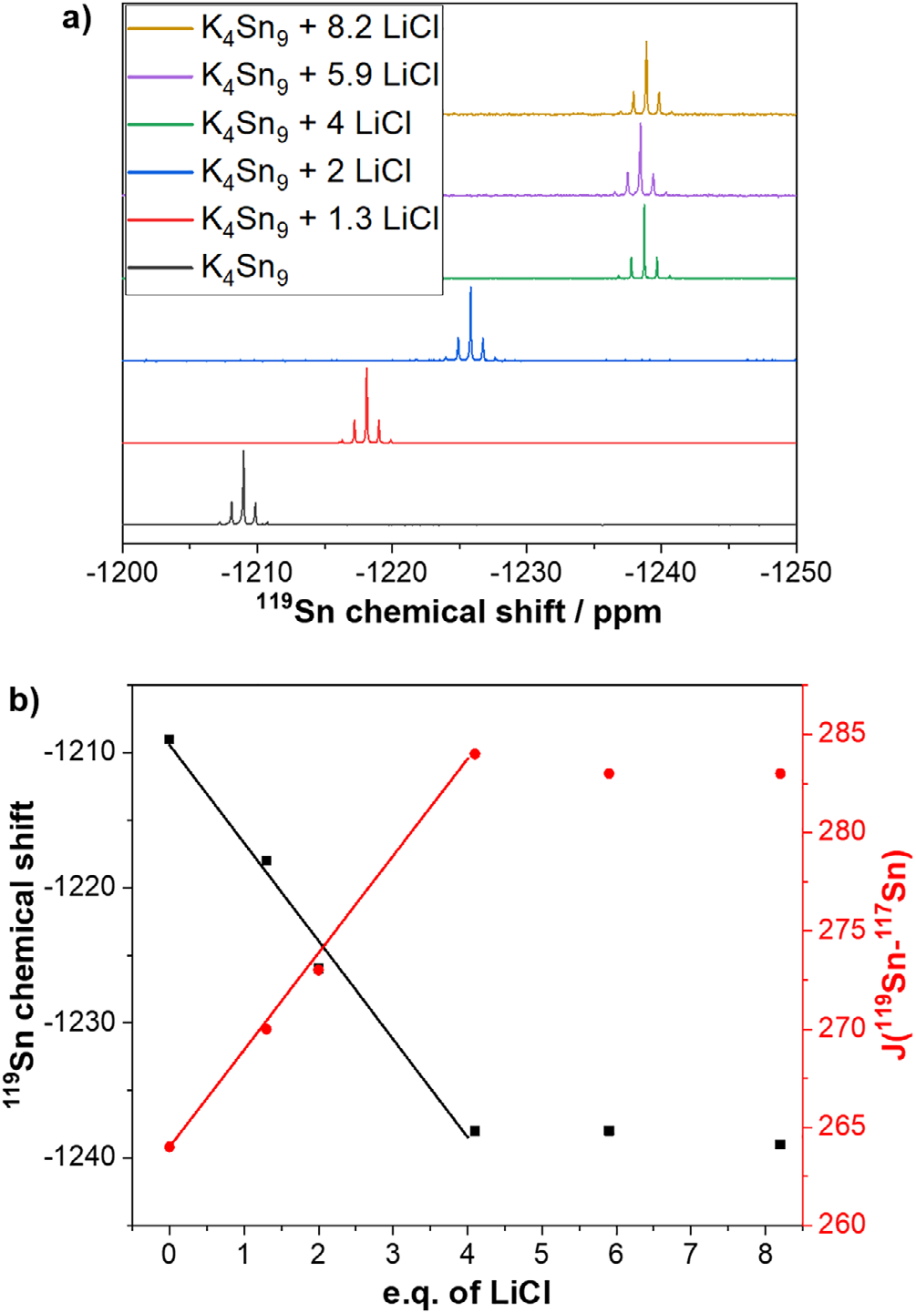
a) Influence of the ^119^Sn chemical shift by adding varying amounts of LiCl to K_4_Sn_9_ in *en*. b) Linear trends for the ^119^Sn chemical shift and ^117^Sn‐^119^Sn coupling constant plotted over the number of equivalents of LiCl added to a solution of K_4_Sn_9_.

To further investigate the influence of the Li^+^ cations on the chemical shift and the coupling constant of the ^119^Sn NMR signals, variable amounts of LiCl were added to K_4_Sn_9_ in *en* solutions (Figure 5).

Addition of stoichiometric amounts of solid LiCl to K_4_Sn_9_ dissolved in *en* results in precipitation of K^+^ ions as KCl and consequently formation of [Sn_9_]^4−^ with mixed counter cations Li*_x_* K_(4−_ *_x_*_)_Sn_9_. The amount of added LiCl to K_4_Sn_9_ shows a linear relationship with the low field shifted NMR signals of the tin cluster (Li*_x_* K_(4−_ *_x_*_)_Sn_9_) from −1209 ppm (for *x* = 0) to −1238 ppm (for *x* = 4) and similarly an increase of the ^117^Sn‐^119^Sn coupling constant from 264 Hz (for *x* = 0) to 284 Hz for (*x* = 4) (Figure 5). Addition of an excess of six or eight equivalents of LiCl to K_4_Sn_9_ does not significantly change the chemical shift or coupling constant, therefore solvation effects can be ruled out as the source for the increase in the coupling constant or upfield shift. An increase in the coupling constant in the series Li*_x_* K_(4−_ *_x_*_)_Sn_9_ suggests either a change of the Sn─Sn bond lengths or a change in the electronic environment of anion **2** with increasing *x*. On one hand polyhedral [Sn_9_]^4−^ clusters are known for significant structural deviations in the solid state and high dynamics in solution, on the other hand Sn–Sn interactions seem to have a negligible influence on these structural variations.^[^ ^80^ ^]^ For example, comparing a similar tin cluster with potassium counterions ([K(18‐crown‐6)]_2_K_2_[Sn_9_]) to anion **2** shows nearly identical bond lengths. In the former, bond lengths range from 2.927(1) to 3.264(1) Å,^[^ ^81^ ^]^ while in anion **2** they range from 2.9393(7) and 3.2722(6) Å. The cluster volumes are also comparable at 33.000 and 33.007 Å^3^, respectively, indicating no significant elongation of the Sn─Sn bonds. Therefore, the increase in the coupling constant is interpreted as originating from different electronic interactions between the K^+^ and Li^+^ counterions with the anionic cluster and the solvent. In principle, different interactions of cations with the cluster anion can influence the fluxional cluster dynamic on the NMR time scale, meaning that a faster rearrangement leads to the observed larger coupling constant with an increased Li^+^/K^+^ ion ratio. As pointed out above, solvent separated ion pairs are more likely for Li^+^ ions thashow coordination to *en* in all compounds **1** to **5**. This is supported by the fact, that the ^7^Li NMR chemical shift of Li_(4−_ *_x_*_)_K*_x_* Sn_9_ at 1.7 ppm is independent of the Li^+^ ion concentration (Figure S8). Additionally, for Li^+^/[Sn_9_]^4−^ contact ion pairs a shift of the ^7^Li NMR signal is expected but was not observed. Furthermore, the observed change on the ^119^Sn NMR shift for [Sn_9_]^4−^ anions with mixed‐counterions is in good agreement with previous findings on the influence of clusters with single‐type counterions *A*_4_Sn_9_ (*A* = Li to Cs).^[^ ^44^ ^]^ Similarly, Eichhorn et al. reported a chemical shift and signal broadening in ^119^Sn NMR spectra of K_4_Sn_9_ dissolved in *en* upon the addition of cryptand.^[^ ^82^ ^]^ The observed increase in chemical shift in all three ion systems (single‐type counterions, sequestered counterions, and mixed counterions) can be best described as a change from ion pairs to separated ion pairs.

Last, the conclusions drawn from NMR spectroscopy are supported by the crystal structures of compounds **1**–**5**. The role of the ions is made particularly clear by the arrangement of the cations in **4**. While the K^+^ ions are in close proximity to the cluster anions (Figure 4b), Li^+^ ions are fully coordinated by the solvent molecules, avoiding direct Li−Sn atom contacts (Figure 4d). Alkali‐metal cations are usually expected to be chemically inert, however, an increase in ion pairing (with increased alkali‐metal radius as shown before) can reduce charge density and therefore also increase the stability of the anionic cluster. This makes them interesting molecules for follow‐up reactions.

## 3 Conclusion

In summary a series of *Zintl* clusters with Li^+^ counterions was synthesized by salt metathesis reactions. Raman spectroscopy of compounds **1** and **2** shows typical bands of [*E*_9_]^4−^, indicating an independence of the vibration modes of the cationic environment. Solvated Li^+^ ions were observed by ^7^Li NMR for compounds **1** to **5**. The ^119^Sn NMR spectra of [Sn_9_]^4−^ reveal a cation dependence of the chemical shift and the coupling constant, which strongly hints for K^+^/[Sn_9_]^4−^ ion pairing, which is absent for Li^+^ counterions that coordinate to *en* molecules. This shows that not all alkali‐metal cations are fully solvated and that the ions play an important role in compensation of the high cluster charge. A more precise fine‐tuning and understanding of the reactivity of anionic clusters in *en* will allow for the formation of large and well‐defined cluster units.

## 4 Experimental Procedures

### General considerations

All experiments were performed under a dry and oxygen‐free argon atmosphere using standard glovebox and Schlenk techniques. The glassware was dried by heating it at 550°C in vacuo. Ethylenediamine (Sigma Aldrich) was dried by refluxing over calcium hydride for 72 h, and anhydrous lithium chloride was dried over sulfuryl chloride and afterwards annealed at 400 °C in dynamic vacuum for 8 h, grinded into a fine powder. Toluene (VWR) was purified by an MBraun solvent purification system, degassed by freeze‐pump‐thaw method, and stored over 3 Å molecular sieves (Merck). The solid phases with the nominal composition K_4_ *E*_4_ and K_4_ *E*_9_ were prepared by the fusion of stoichiometric amounts of the elements at elevated temperatures in tantalum ampules or in a steel autoclave according to the literature.^[^ ^7^, ^48^, ^51^ ^]^ A qualitative water test for the solvent *en* was done according to the literature. In brief, the solid 1,4‐*bis*(trimethylsilyl)butadiyne (93.4 mg, 480 µmol) was weighed out in a Schlenk tube, and 2 mL of *en* was added. The reaction was stirred for 20 h, and the residual unreacted bis(trimethylsilyl)butadiyne was filtered off prior to characterization.^[^ ^83^ ^]^

### NMR spectroscopy

^1^H NMR spectra were measured on a Bruker Avance Ultrashield 400 MHz spectrometer; the ^7^Li, ^119^Sn, and ^207^Pb spectra were measured on a  Bruker Avance 300 MHz spectrometer. ^1^H NMR spectra were acquired using a relaxation delay of 5 s, an acquisition time of 4 s, and a total of 16 scans, with a spectral window of 20 ppm. ^7^Li NMR spectra were collected under similar conditions, with a relaxation delay of 5 s, an acquisition time of 2 s, and 64 scans, using a spectral window of 90 ppm. For ^119^Sn NMR, the relaxation delay ranged from 0.3 to 1 s, with an acquisition time between 0.3 and 0.4 s. The number of scans varied from 10,000 to 45,000, and the spectral window was set to 500 ppm. Similarly, ^207^Pb NMR spectra were obtained with a relaxation delay of 0.1 s, an acquisition time of 1.7 s, and a total of 10,000 scans, using a spectral window of 500 ppm. The ^1^H NMR spectra were calibrated using the solvent signals. An internal, airtight capillary filled with deuterated solvent (C_6_D_6_) was used for locking the samples. The ^7^Li, ^119^Sn, and ^209^Pb are referenced against an external standard 9.7 M LiCl in D_2_O (0 ppm), Me_4_Sn in C_6_D_6_ (0 ppm), and Pb(NO_3_)_2_ in D_2_O (−2961.2 ppm), respectively. Unless stated otherwise, the molar concentration of the cluster dissolved in *en* for the NMR experiments is approximately 76 mM. Singlets are abbreviated with (s). MestReNova was used for evaluating the spectra.

### Energy‐dispersive X‐ray spectroscopy

Several single crystals of **1** to **5** were mounted on carbon tape and were analyzed with a scanning electron microscope equipped with an energy dispersive X‐ray analyzer (Hitachi TM‐1000 Tabletop microscope).

### Powder X‐ray diffraction

For powder X‐ray diffraction (PXRD) analysis the samples were finely ground in an agate mortar, diluted with diamond powder (for *E* = Pb) and sealed in glass capillaries (outer diameter 0.3 mm, wall thickness 0.01 mm, MARK capillaries, Müller & Müller OHG) with capillary wax (Hampton Research) in an argon‐filled glove box. PXRD were performed at room temperature by using a Stoe STADI P powder diffractometer equipped with a linear position‐sensitive detector (Mythen 1K) using Cu *K*_α1_ (*λ* = 1.54060 Å) or Mo *K*_α1_ (*λ* = 0.70926 Å) radiation and curved Ge (111) monochromators. The samples were measured in Debye–Scherrer geometry (2*θ*_max_ = 45°). Data analysis was carried out by using the Stoe WinXPOW software package.^[^ ^84^ ^]^

### Raman spectroscopy

Raman spectroscopic measurements were performed using a Renishaw inVia Raman microscope equipped with a CCD detector and laser (785 or 520 nm) with a maximum power of 0.01 mW. The samples were measured for 100 s. For operating the device, the software WiRe 4.2 was used. Samples were ground in an agate mortar or single crystals picked inside of a glove box and then filled into glass capillaries (outer diameter 0.3 mm, wall thickness 0.01 mm, MARK capillaries, Müller & Müller OHG), which were sealed using capillary wax (Hampton Research).

### IR spectroscopy

FT‐IR spectra were recorded on a Spectrum Two (UATR TWO, Perkin Elmer) spectrometer with a diamond sensor. To avoid water and oxygen contamination, the device is operated inside an argon‐filled glovebox. Measurements are taken between 4000 and 500 cm^−1^ with a spectral resolution of 1 cm^−1^.

### Single crystal structure determination

The air‐ and moisture‐sensitive crystals of **1**, **2**, **3**, **4**, and **5** were transferred from the mother liquor into perfluoroalkyl ether oil and isolated in a Glovebox. For diffraction data collection, the single crystals were mounted on a glass capillary and positioned in a 150 K cold N_2_ gas stream. Data collection was performed with a STOE StadiVari diffractometer (Mo *K_α_* radiation) equipped with a DECTRIS PILATUS 300K detector. Structures were solved by Direct Methods (SHELXS‐97) and refined by full‐matrix least‐squares calculations against *F* ^2^ (SHELXL‐2014 or SHELXL‐2018).^[^ ^85^ ^]^ The positions of the hydrogen atoms were calculated and refined using a riding model. Unless stated otherwise, all non‐hydrogen atoms were treated with anisotropic displacement parameters. Pictures of the crystal structures were created with the program Diamond.^[^ ^86^ ^]^ Details of the crystallographic data for compounds **1** to **5** are summarized in Tables S4 and S5. Further details of the crystal structure investigations may be obtained from the joint CCDC/FIZ Karlsruhe online deposition service via www.ccdc.cam.ac.uk/data_request/cif, on quoting the depository numbers CCDC‐2289462 – 2,289,466.

### Polynator

The volume of nine‐atomic clusters was calculated using the program polynator.^[^ ^87^ ^]^ For determining the molecular connectivity of the cluster framework, the radius of Sn was set to 2 Å. The crystallographic information file (CIF) used for this analysis was obtained from the Cambridge Crystallographic Data Centre.^[^ ^81^ ^]^

### Synthesis and crystallization of [Li(*en*)_2.5_]_4_[Ge_9_] (1)

Solid K_4_Ge_4_ (67.7 mg, 150 µmol, and 1equiv.) and LiCl (25.4 mg, 600 µmol, and 4 equiv.) were weighed into a Schlenk tube and *en* (2 mL) was added. The red solution was stirred for 3 h, filtered and layered with 2 mL toluene for crystallization. Overnight, orange block shaped crystals suitable for single crystal diffraction formed (59.0 mg, 69%). EDX measurements confirmed the presence of Ge and the absence of K.

**^7^** Li NMR (400 MHz, 298 K, *en*/C_6_D_6_): δ [ppm] = 1.70 ppm. Raman: 80, 114, 142, 166, and 228 cm^−1^. EDX measurements confirmed the absence of K and the presence of Ge.

### Synthesis and crystallization of [Li(*en*)_2_]_4_[Sn_9_] (2)

Solid K_4_Sn_4_ (94.7 mg, 150 µmol, and 1equiv.) and LiCl (25.4 mg, 600 µmol, and 4 equiv.) were weighed into a Schlenk tube, and *en* (2 mL) was added. The deep red solution was stirred for 3 h, filtered, and layered with 2 mL toluene for crystallization. After a week, dark red block crystals suitable for single crystal diffraction formed (44.0 mg, 42%). EDX measurements confirmed the presence of Sn and the absence of K.

**^7^** Li NMR (400 MHz, 298 K, *en*/C_6_D_6_): δ [ppm] = 1.7 ppm. ^119^Sn NMR (400 MHz, 298 K, *en*/C_6_D_6_): δ [ppm] = −1238 (s, ^1^ *J* ^117^_Sn‐_^119^_Sn_ = 283 Hz). Raman: 156 cm^−1^. EDX measurements confirmed the absence of K and the presence of Sn.

### Synthesis and crystallization of [Li(*en*)_2_]_4_[Pb_9_] (3)

Solid K_4_Pb_4_ (147.8 mg, 150 µmol, and 1 equiv.) and LiCl (50.8 mg, 1.2 mmol, and 8 equiv.) were weighed into a Schlenk tube and *en* (2 mL) was added. The deep reddish‐brown solution was stirred for 3 h, filtered, and layered with 2 mL toluene for crystallization. After 3 days, dark red octahedral‐shaped crystals suitable for single crystal diffraction besides colorless needles of Li(en)_2_Cl formed. EDX measurements confirmed the presence of Pb and the absence of K.

^7^Li NMR (400 MHz, 298 K, *en*/C_6_D_6_): δ [ppm] = 1.71 ppm. ^119^Pb NMR (400 MHz, 298 K, *en*/C_6_D_6_): δ [ppm] = −4192. EDX measurements confirmed the absence of K and the presence of Pb.

### Synthesis and crystallization of [Li_6_(*en*)_13_][Ge_9_‐Ge_9_] (4)

Solid K_4_Ge_9_ (121.5 mg, 150 µmol, and 1 equiv.) was weighed out in a Schlenk tube and dissolved in 2 mL *en*. To the resulting deep red solution, LiCl (25.4 mg, 600 µmol, and 4 equiv.) was added. After stirring for 3 h, the red solution was filtered and subsequently, the filtrate was carefully layered with 2 mL toluene. After 1 week, deep red needle‐shaped crystals of compound **4** formed. EDX measurements confirmed the presence of Ge.

^7^Li NMR (400 MHz, 298 K, *en*/C_6_D_6_): δ [ppm] = 1.71 ppm. EDX measurements confirmed the absence of K and the presence of Ge.

### Synthesis and crystallization of [Li(*en*)_2_]_4_K_2_[Ge_9_‐Ge_9_] (5)

Solid K_4_Ge_9_ (121.5 mg, 150 µmol, and 1 equiv.) was weighed out in a Schlenk tube and dissolved in 2 mL *en*. To the resulting deep red solution, LiCl (12.7 mg, 300 µmol, and 2 equiv.) was added. After stirring for 3 h, the red solution was filtered, and subsequently, the filtrate was carefully layered with 2 mL toluene. After 1 week, deep red block‐shaped crystals of compound **5** formed. EDX measurements confirmed the presence of K and Ge.

^7^Li NMR (400 MHz, 298 K, *en*/C_6_D_6_): δ [ppm] = 1.71 ppm. EDX measurements qualitatively confirmed the presence of K and Ge.

### NMR spectroscopic investigation on the influence of LiCl and K_4_Sn_9_

Prior to the addition of 2 mL *en*, the solids K_4_Sn_9_ and LiCl were weighed out in a Schlenk tube in the different ratios (Table S1). The mixture was stirred for 1 h, filtered and 0.5 mL of the filtered solution was transferred to the NMR tube for analysis.

## Supporting Information

The authors have cited additional references within the Supporting Information.^[^ ^81^, ^83^, ^88^, ^89^, ^90^, ^91^ ^]^

## Conflict of Interests

The authors declare no conflicts of interest.

## Supporting information



Supporting Information

## Data Availability

The data that support the findings of this study are available from the corresponding author upon reasonable request.
