# Supplementary material for: The Role of Lithium Ions on the Solubility of K4 E 4 in Ethylenediamine and the Oxidation of the Zintl Anions [E4]4− (E = Ge, Sn, Pb) as well as [Ge9]4−
Source: Chemistry. 2025 Apr 8;31(26):e202500592. doi: 10.1002/chem.202500592 (PMC12063050; doi:10.1002/chem.202500592)
Supplement: Supplementary file 1 — Supporting Information [file CHEM-31-e202500592-s001.docx]

Supporting Information
©Wiley-VCH 2021
69451 Weinheim, Germany

The Role of Lithium Ions on the Solubility of K_4_*E*_4_ in Ethylenediamine and the Oxidation of the *Zintl* Anions [*E*_4_]^4-^ (*E* = Ge, Sn, Pb) as well as [Ge_9_]^4-^

Christian E. Fajman, Dominik M. Dankert and Wilhelm Klein and Thomas F. Fässler*

**Table of Contents**

[1. Experimental Procedures 3](#_Toc189561743)

[2. ^7^Li, ^119^Sn and ^207^Pb NMR 4](#_Toc189561744)

[3. Raman Spectroscopy 11](#_Toc189561745)

[4. EDX Analysis 12](#_Toc189561746)

[5. Characterization of Starting Materials and Side Products 13](#_Toc189561747)

[5.1. Purity of Ethylenediamine 13](#_Toc189561748)

[5.2. PXRD of *Zintl* Phases 14](#_Toc189561749)

[5.3. Raman of *Zintl* Phases 15](#_Toc189561750)

[5.4. PXRD and FTIR of LiCl 15](#_Toc189561751)

[5.5. PXRD of the Precipitate 16](#_Toc189561752)

[6. Crystallographic Data 17](#_Toc189561753)

[References 32](#_Toc189561754)

[Author Contributions 32](#_Toc189561755)

1. Experimental Procedures

**NMR spectroscopic investigation on the influence of LiCl and K_4_Sn_9_**

**Table S1**: The mass and amount of substance of K_4_Sn_9_ and LiCl are given. From the amount of substance, a molar ratio was calculated, which gives the number of equivalents of LiCl per tin cluster.

| **m(K_4_Sn_9_) / mg** | **m(LiCl) / mg** | **n(K_4_Sn_9_) / µmol** | **n(LiCl) / µmol** | **ratio (n(LiCl)/n(K_4_Sn_9_))** | |
| --- | --- | --- | --- | --- | --- |
| 46.8 | 13.2 | 38.2 | 311.4 | 8.2 |  |
| 59.6 | 12.2 | 48.7 | 287.8 | 5.9 |  |
| 93.0 | 13 | 75.9 | 306.6 | 4.0 |  |
| 183.7 | 12.8 | 150.0 | 301.9 | 2.0 |  |
| 282.6 | 12.6 | 230.8 | 297.2 | 1.3 |  |
| 184.6 | - | 150.7 | - | - |  |

2. ^7^Li, ^119^Sn and ^207^Pb NMR


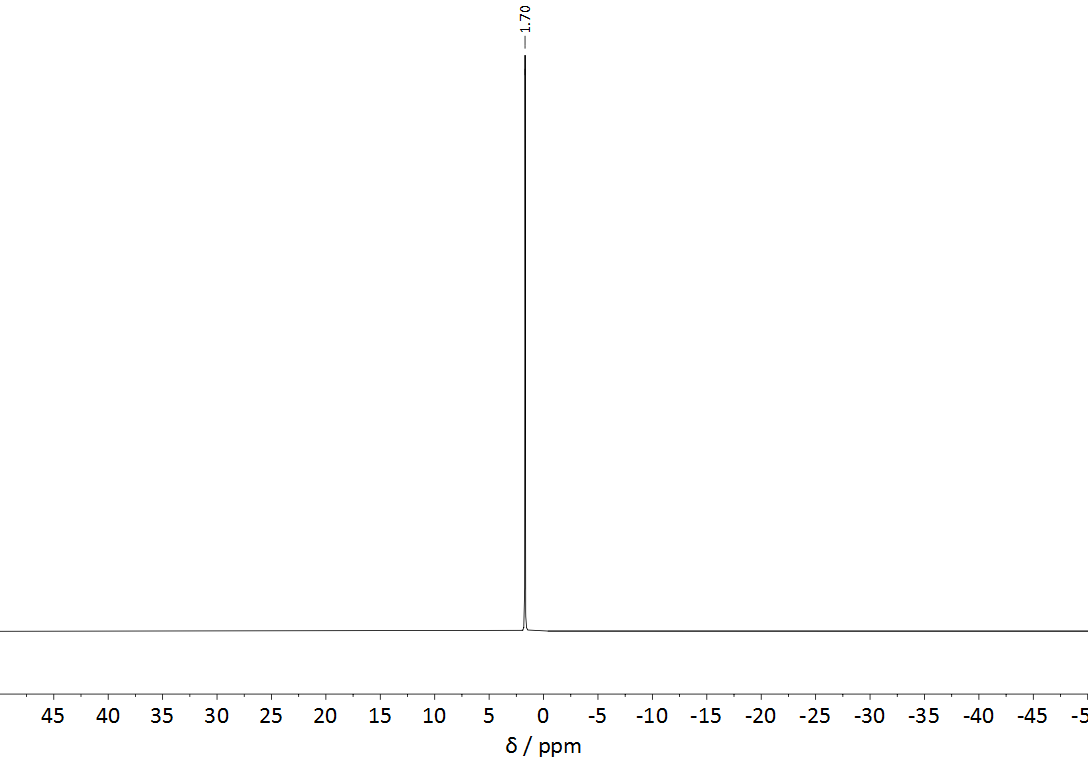

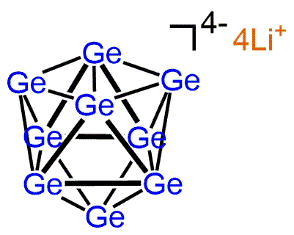


**Figure S1:** ^7^Li NMR spectrum of compound **1** in *en*.


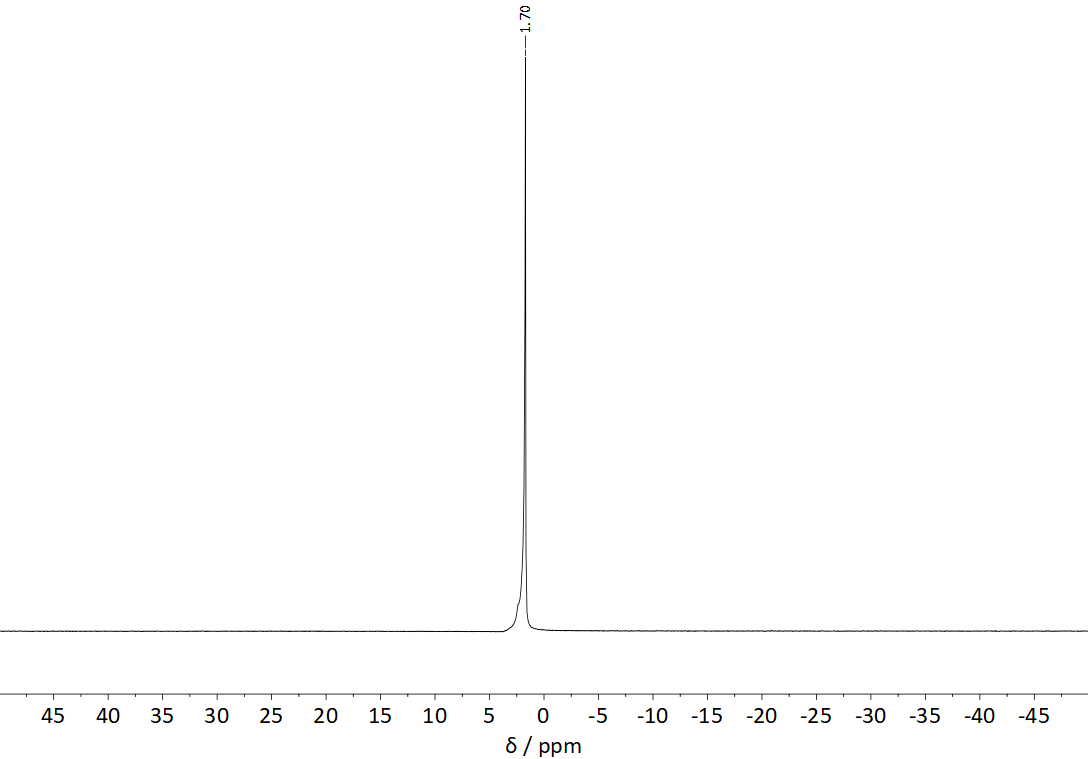

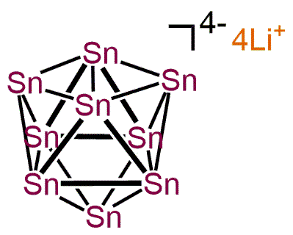


**Figure S2**: ^7^Li NMR spectrum of compound **2** in *en*.


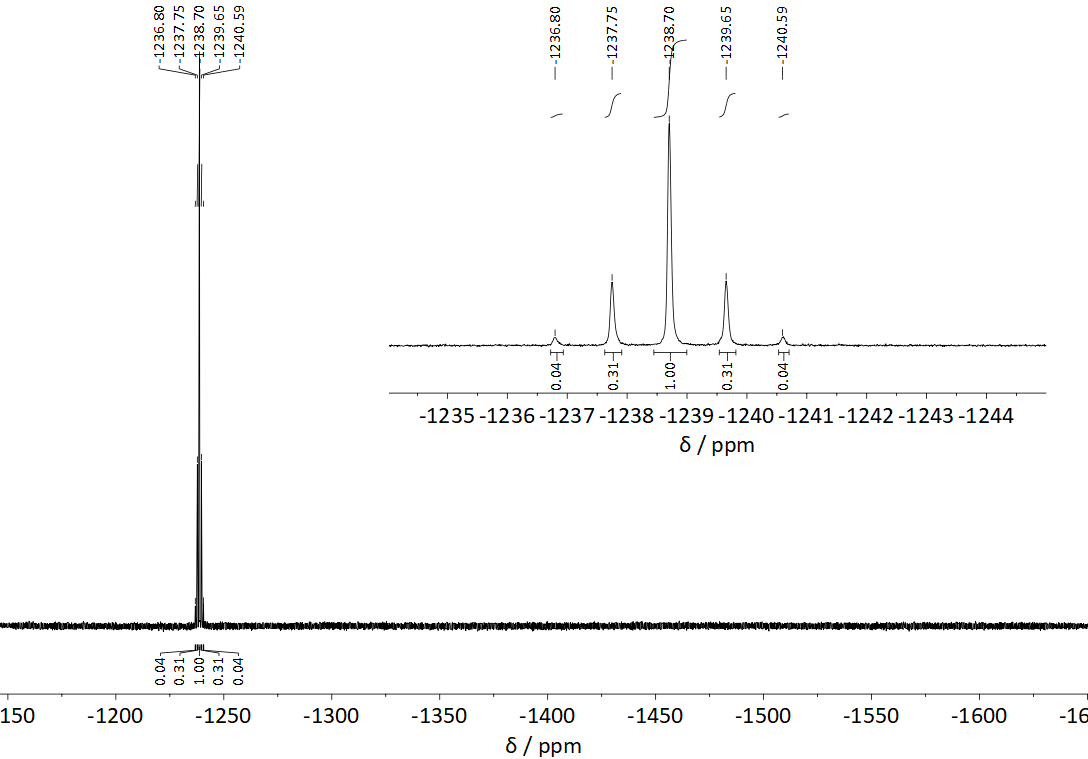

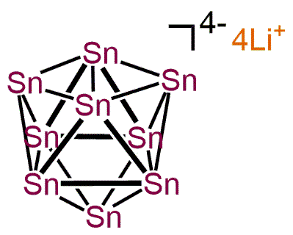


Figure S3: ^119^Sn NMR spectrum of 2 in *en*. The ^119^Sn-^117^Sn coupling constant of 283 Hz, and the relative peak heights of (1:0.31:0.04) matches the calculated (^119^Sn-^117^Sn) isotope pattern for [Sn_9_]^4-^.


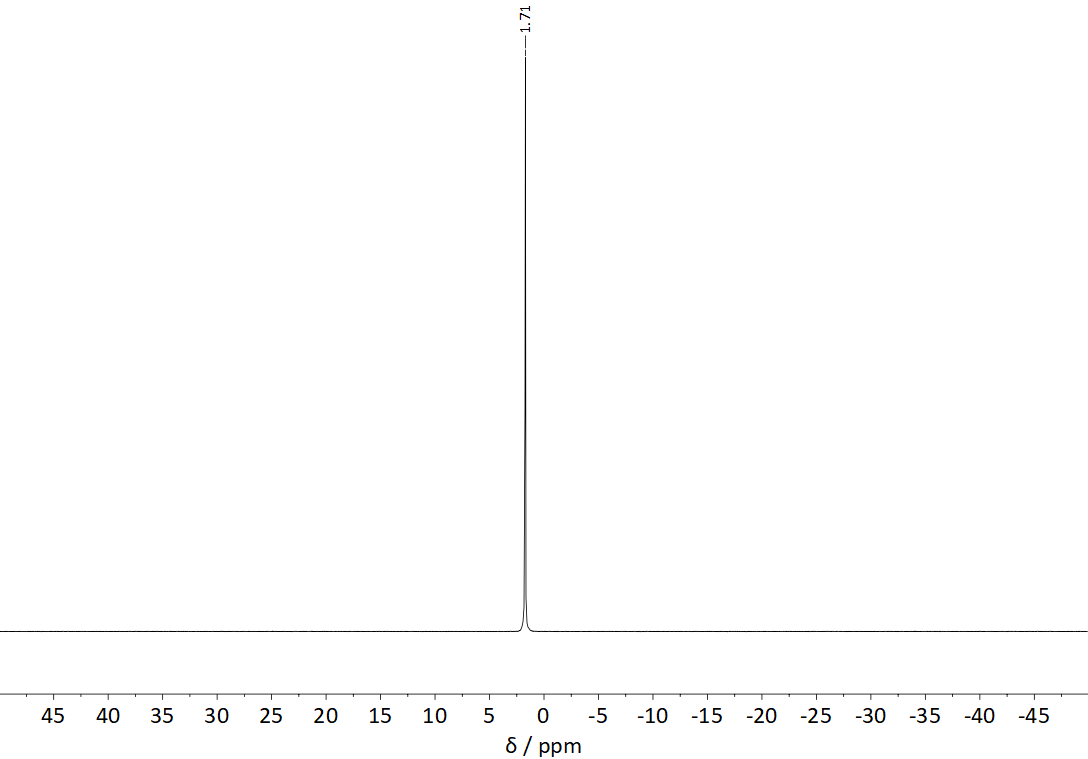

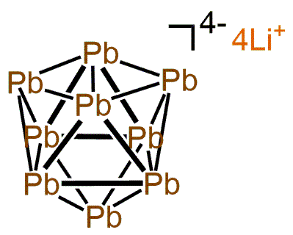


Figure S4: ^7^Li NMR spectrum of compound 3 in *en*.


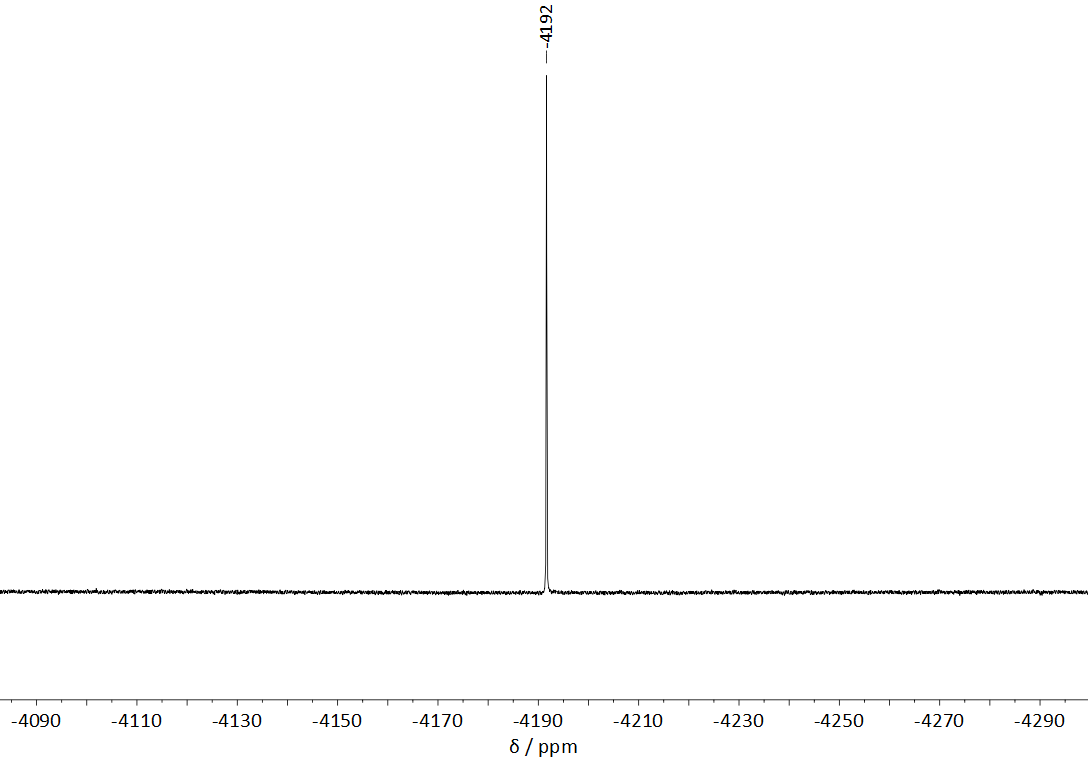

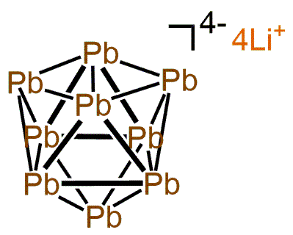


**Figure S5**: ^207^Pb NMR spectrum of compound **3** in *en*.


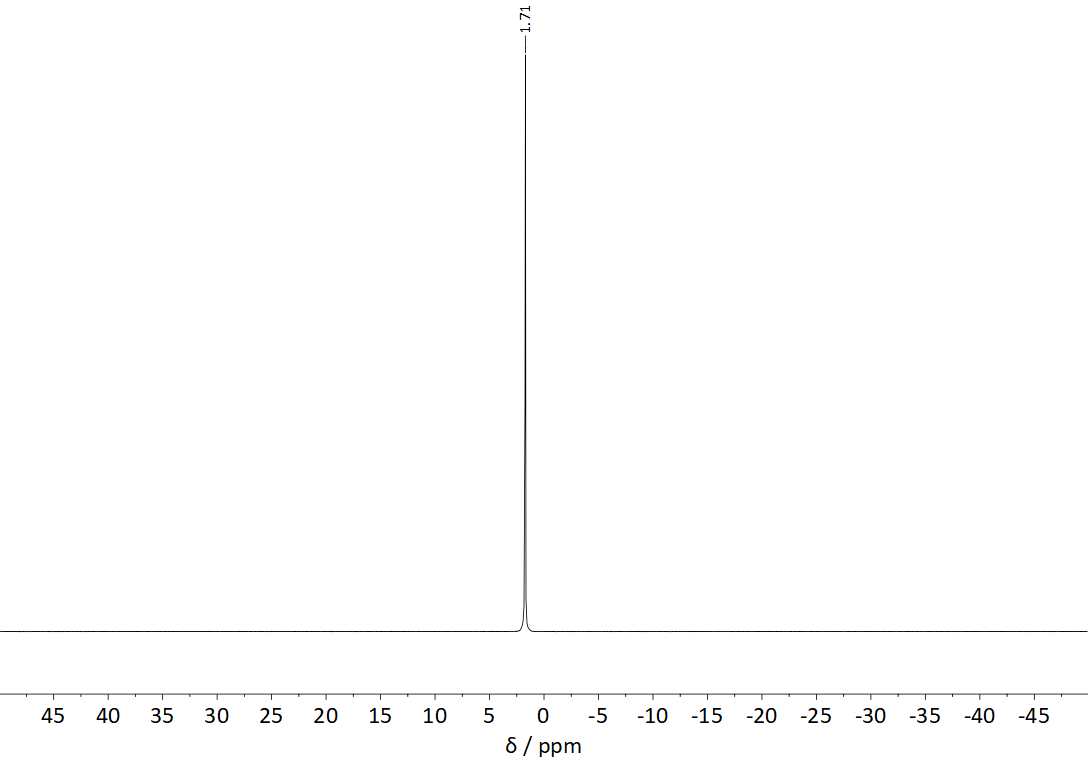

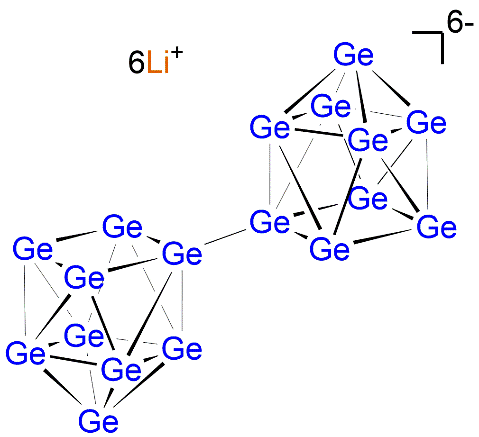


Figure S6: ^7^Li NMR spectrum of compound 4 in *en*.


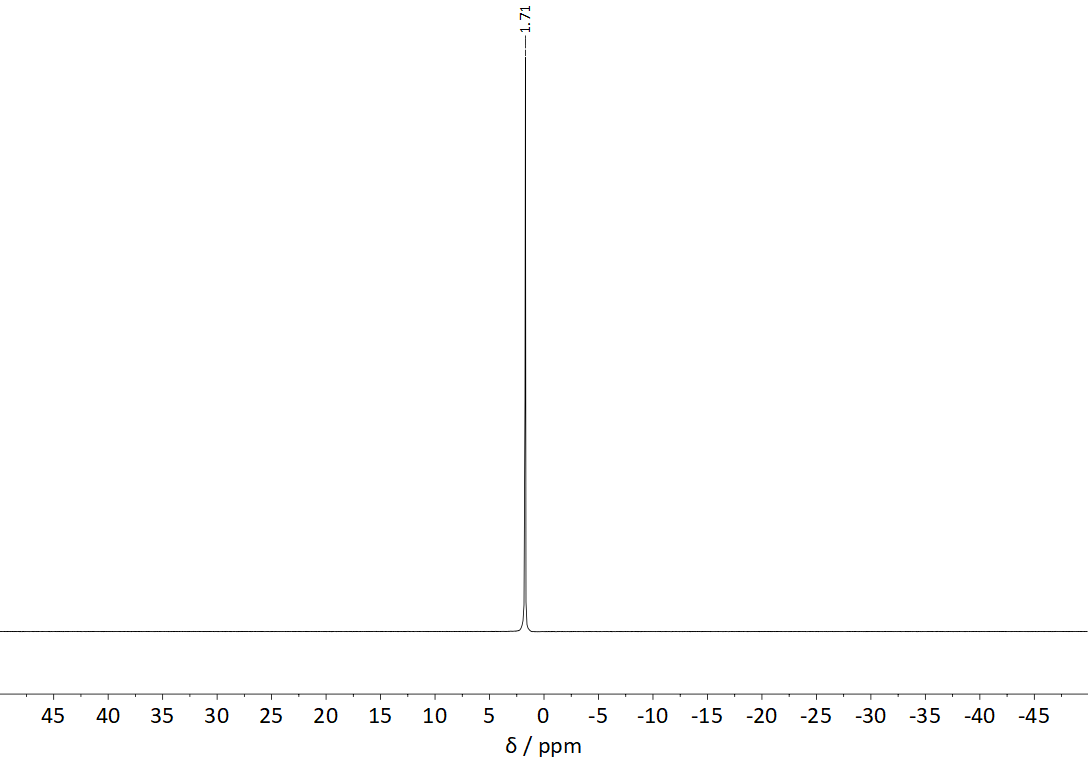

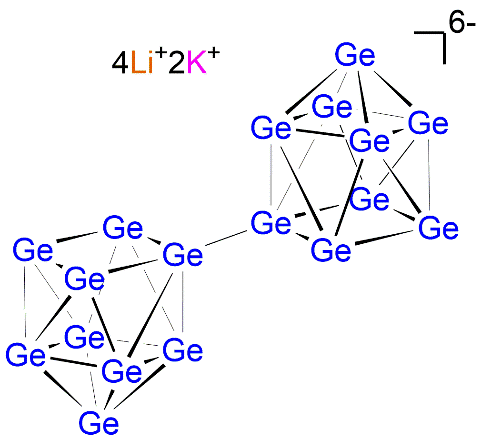


**Figure S7**: ^7^Li NMR spectrum of compound **5** in *en*.


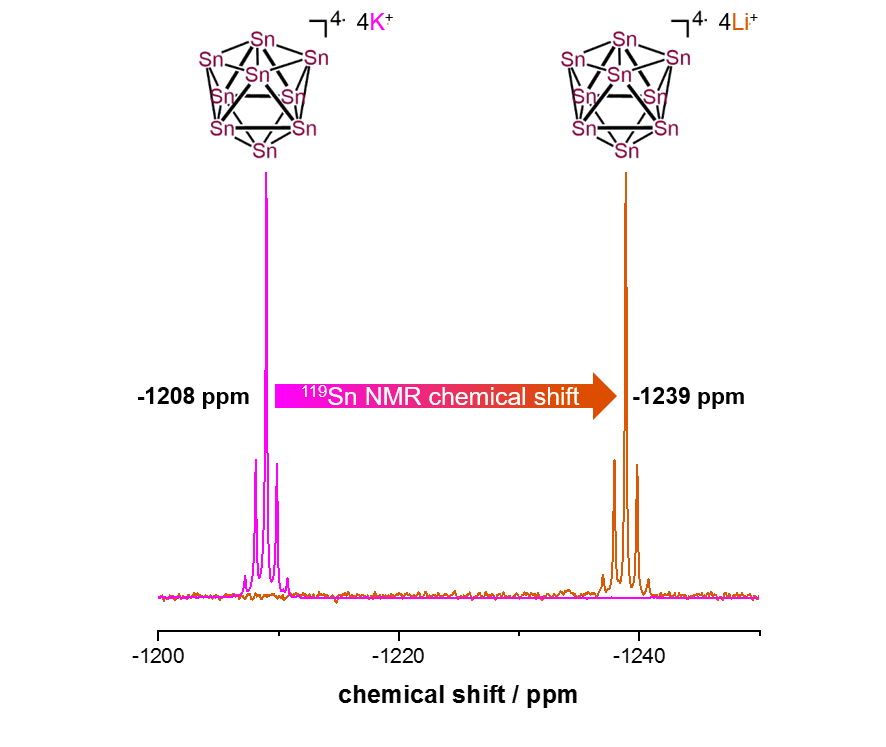


**Figure S8**: Superimposed ^119^Sn NMR spectra of [Sn_9_]^4-^ with Li^+^ and K^+^ counterions in orange and pink, respectively.


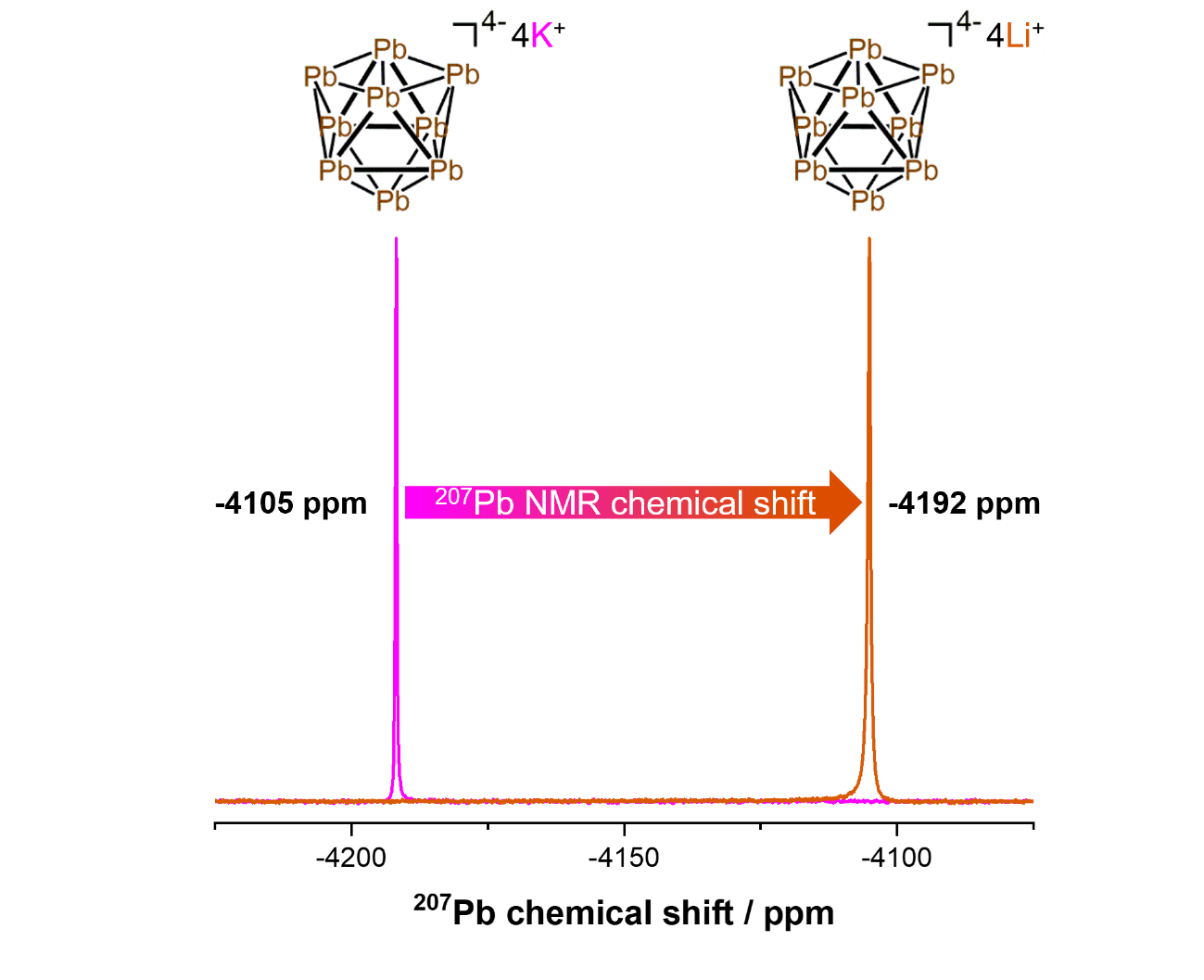


**Figure S9**: Superimposed ^207^Pb NMR spectra of Pb_9_]^4-^ with Li^+^ and K^+^ counterions in orange and pink, respectively.


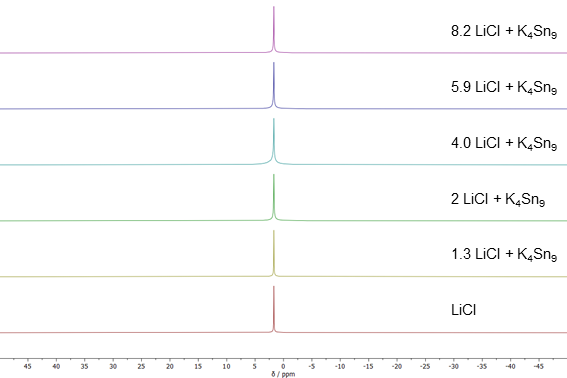


**Figure S10**: Stacked ^7^Li NMR spectra of LiCl and K_4_Sn_9_ in varying ratios in *en*.


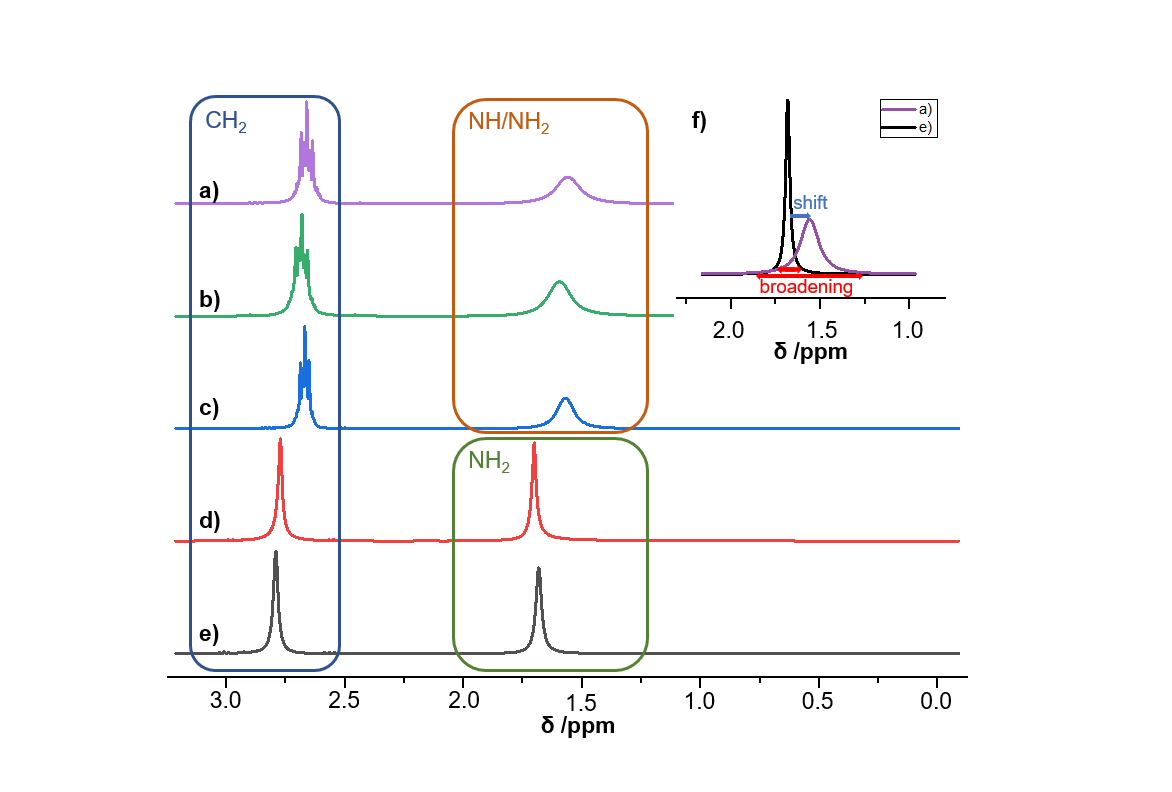


**Figure S11**: Stacked section of the ^1^H NMR spectra of the reaction solution of a) K_4_Ge_4_ and LiCl in *en*; b) K_4_Sn_4_ and LiCl in *en*; c) K_4_Pb_4_ and LiCl in *en*. And as a reference ^1^H NMR spectra of d) LiCl dissolved in *en*; e) pure *en*. In f) the ^1^H NMR spectra of a) and e) are superimposed, visualizing the chemical shift in blue and the signal broadening of the NH/NH_2_ group. The region of the CH_2_, NH_2_ and NH/NH_2_ groups are highlighted in blue, green and orange, respectively.

**Table S2**: Chemical shifts in ppm obtained from the ^1^H NMR spectra in Figure S11.

| **Spectra** | **Mixture** | **CH_2_** | **NH/NH_2_** |
| --- | --- | --- | --- |
| **a)** | K_4_Ge_4_ and LiCl in *en* | 2.66 | 1.56 |
| **b)** | K_4_Sn_4_ and LiCl in *en* | 2.68 | 1.59 |
| **c)** | K_4_Pb_4_ and LiCl in *en* | 2.67 | 1.57 |
| **d)** | LiCl in *en* | 2.77 | 1.70 |
| **e)** | *en* | 2.79 | 1.68 |

3. Raman Spectroscopy

**Figure 12:** Raman spectra of compound **1** (black) and **2** (red) with the *Zintl* ions [*E*_9_]^4−^ in the range of 50 to 800 cm^−1^. Characteristic modes are labeled with their corresponding Raman shifts.

In the Raman spectra characteristic intensive bands for [*E*_9_]^4−^ in the range of 50 to 250 cm^−1^ are observed. Other intensive bands are not present in the Raman spectra. Two bands are reported for K_4_Sn_9_ (lit.: 99, 146 cm^−1^)^[1]^, but only one is observed for anion **2** at 156 cm^−1^. The detection of the band at 99 cm^−1^ is not possible due to the Rayleigh filter cutting of signals below 110 cm^−1^. For the measurement of compound **1** a different laser with another wavelength was used. Therefore, the Rayleigh filter cuts off at a different wavenumber and allows for the detection of bands below 110 cm^−1^. Measuring the compound **2** with lasers of other wavelength was hampered by weak intensities.

4. EDX Analysis


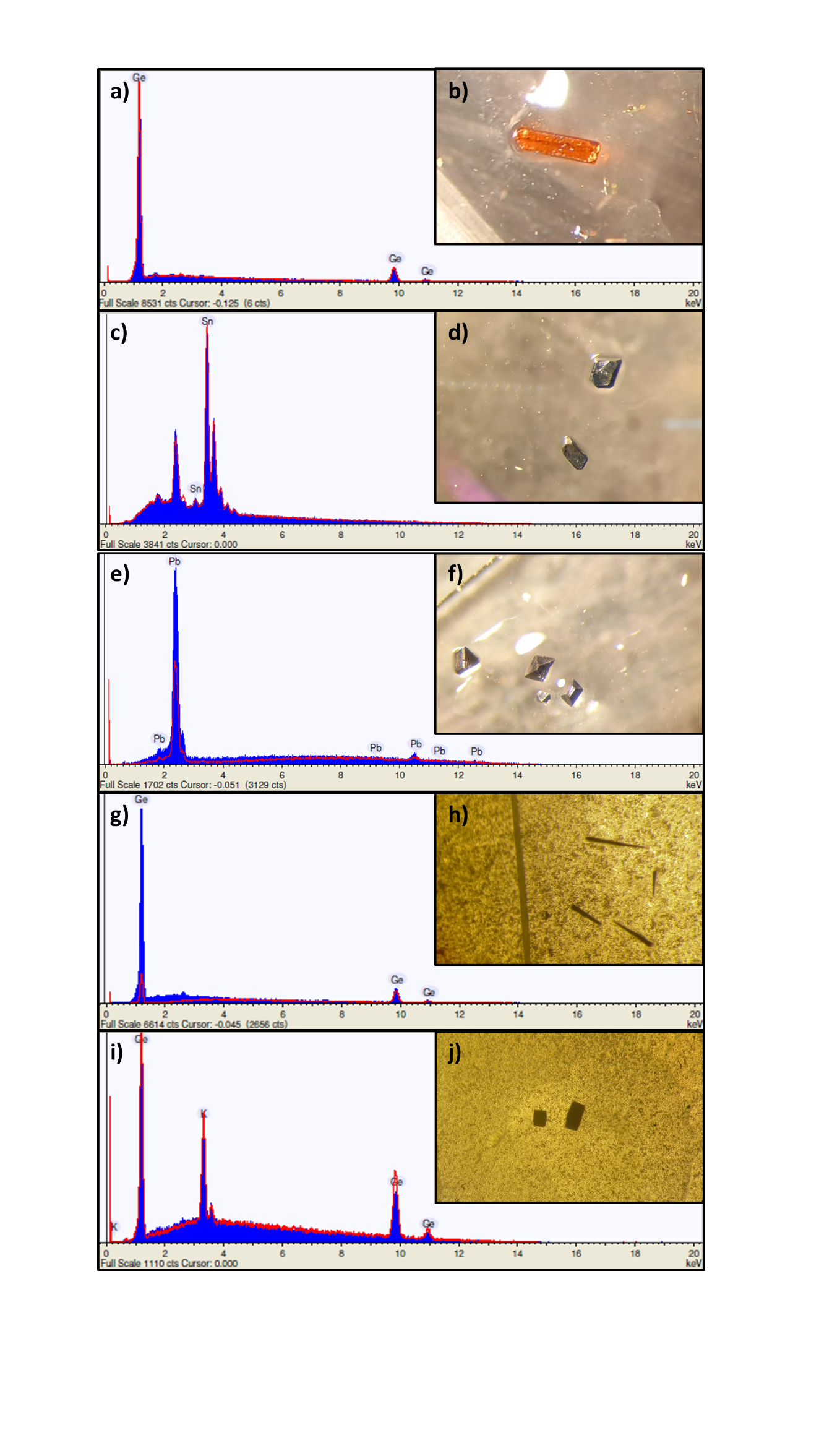


**Figure S13**. EDX analysis of compounds a) **1** c) **2** e) **3** g) **4** i) **5** and pictures of corresponding single crystals in b), d), f), h) and j).

**Table S3**: EDX analysis of compounds **1** to **5** and the amount of elements found and calculated in weight percent.

| **Compound** | **Element** | **Element wt%(exp)** | **Element wt%(calc.)** |
| --- | --- | --- | --- |
| **[Li(en)_2.5_]_4_[Ge_9_] (1)** | Ge | 100 | 100 |
| **[Li(en)_2_]_4_[Sn_9_] (2)** | Sn | 100 | 100 |
| **[Li(en)_2_]_4_[Pb_9_] (3)** | Pb | 100 | 100 |
| **[Li(en)_2_]_6.5_[Ge_9_-Ge_9_] (4)** | Ge | 100 | 100 |
| **[Li(en)_2_]_4_K_2_[Ge_9_-Ge_9_] (5)** | Ge | 75 | 94 |
|  | K | 52 | 6 |

5. Characterization of Starting Materials and Side Products

5.1. Purity of Ethylenediamine

The purity if ethylenediamine can be analysed by a qualitative water test by reacting 1,4‑bis(trimethylsilyl)butadiyne with *en*. The resulting reaction has two possible products (3*Z*)- and (3*E*)-1-trimethylsilyl-7-amino-5-aza-hepta-3-en-1-yne under water-free conditions. In case traces of water are present in *en*, the reaction product reacts further to 2,3-dihydro-5-methyl-1H-1,4-diazepine. Both isomers (3Z)- and (3E)-7-amino-1-(trimethylsilyl)-5-aza-hepta-3-en-1-yne are detected and their corresponding peaks assigned (see Figure S14). Because no peaks attributed to 2,3-dihydro-5-methyl-1H-1,4-diazepine are detected, *en* is considered water-free.^[2]^


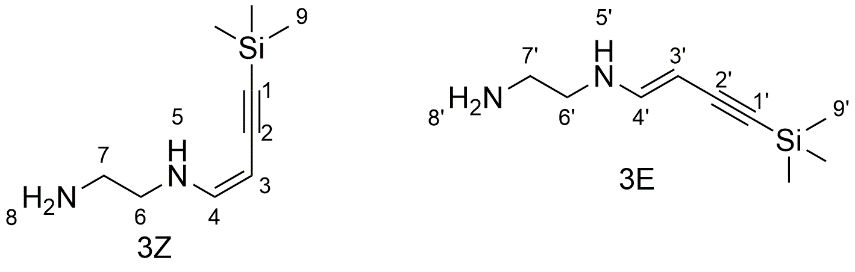

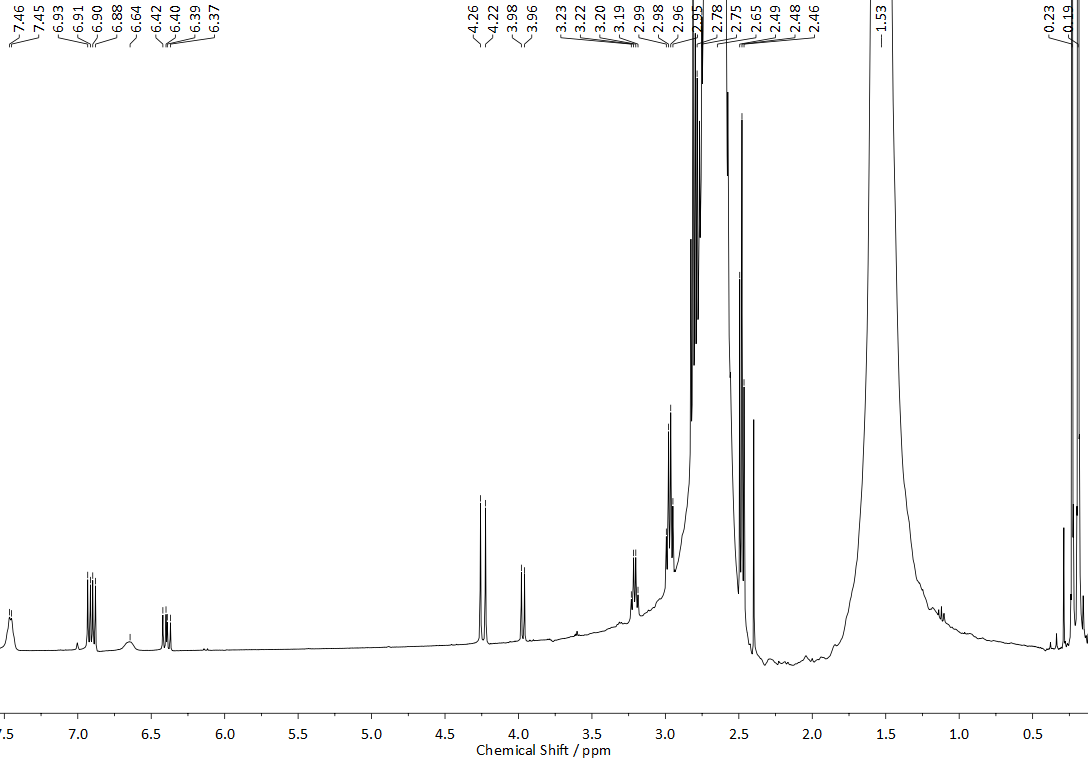


C_6_D_6_

5

7‘

7

en

en

9

6‘

6

3

3‘

4

4‘

5‘

**Figure S14**. ^1^H NMR spectrum after reaction of bis(trimethylsilyl)butadiyne with anhydrous en. A lock capillary with C_6_D_6_ was used. Both isomers (3Z)- and (3E)-7-amino-1-(trimethylsilyl)-5-aza-hepta-3-en-1-yne are detected and their corresponding peaks assigned. Peaks attributed to 8 and 8’ protons are covered by the solvent (en) peaks and can be determined via COSY-NMR.^[2]^

5.2. PXRD of *Zintl* Phases

In the course of the identification of the oxidizing agent, the purity of the starting materials K_4_*E*_4_ were analyzed. The four atomic clusters contain no side phases such as K_4_*E*_9_ or K_12_*E*_17_ according to PXRD (Figure S15) and Raman (Figure S16) for K_4_Ge_4_ and K_4_Sn_4_ (the collection of Raman data for K_4_Pb_4_ was hampered by weak intensities). Therefore, the presence of [*E*_9_]^4-^ cluster in the solid state from the very beginning can be excluded. According to the powder diffraction analysis anhydrous LiCl was observed as the only crystalline phase in the used LiCl. Additionally, the absence of OH vibrations in IR spectra of LiCl indicate that the solid contains no amorphous impurities, which could act as a possible proton source for the oxidation (Figure S17). Finally, the reaction was carried out for 1 h without LiCl. The absence of dissolved clusters was indicated by an almost colorless supernatant solution above the powdered precursor phase. As soon as the LiCl is added to the suspension, the reaction starts and the color vividly changes to deep red, which is typical for solutions containing deltahedral clusters.


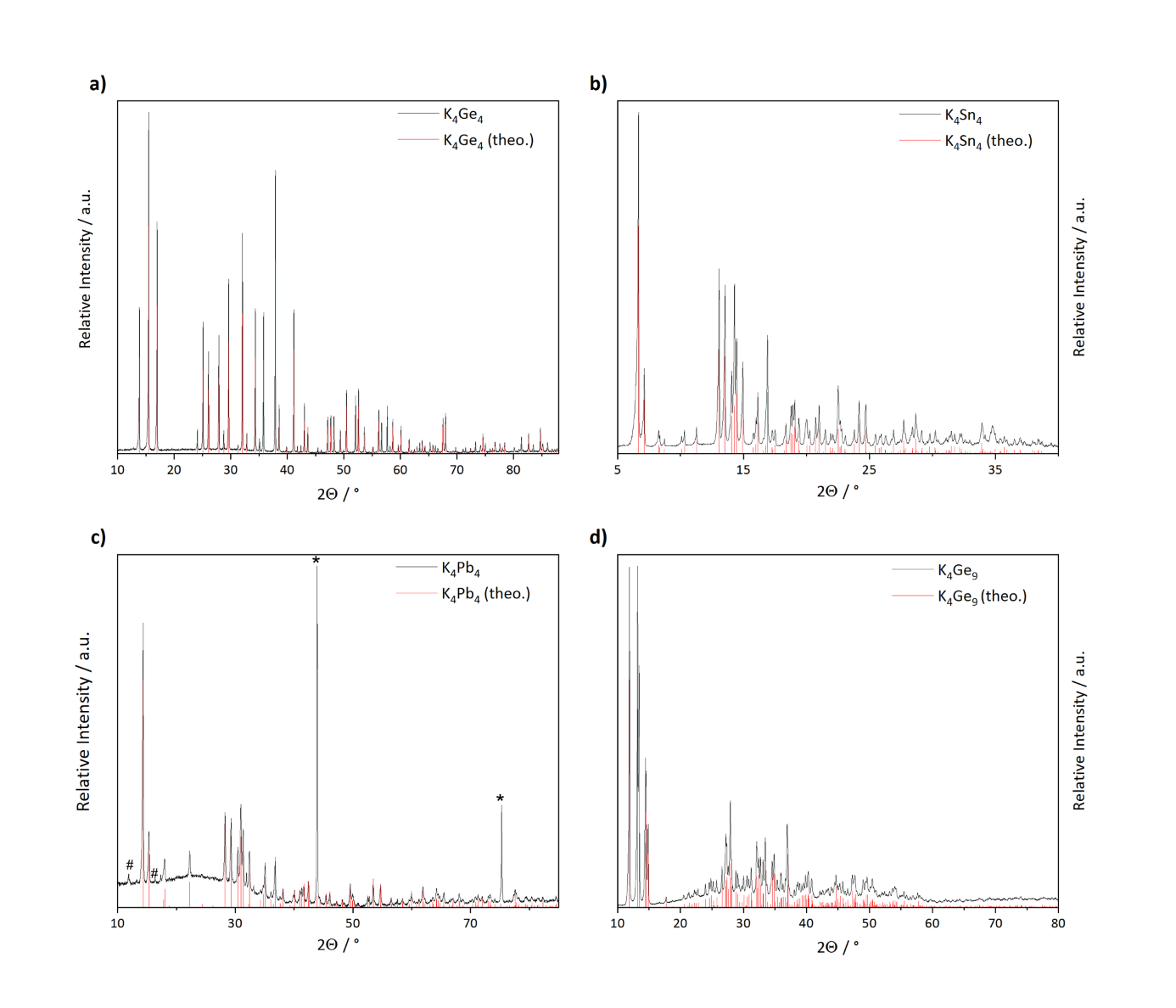


**Figure S15:** Powder X-ray diffraction of *Zintl* clusters with the nominal composition a) K_4_Ge_4_, b) K_4_Sn_4_, c) K_4_Pb_4_, d) K_4_Ge_9_, and their theoretical patterns calculated from single crystal data.^[3-5]^ Reflections of the internal diamond standard are labelled with asterisks. Unidentified reflections are indicated with hash signs.

5.3. Raman of *Zintl* Phases


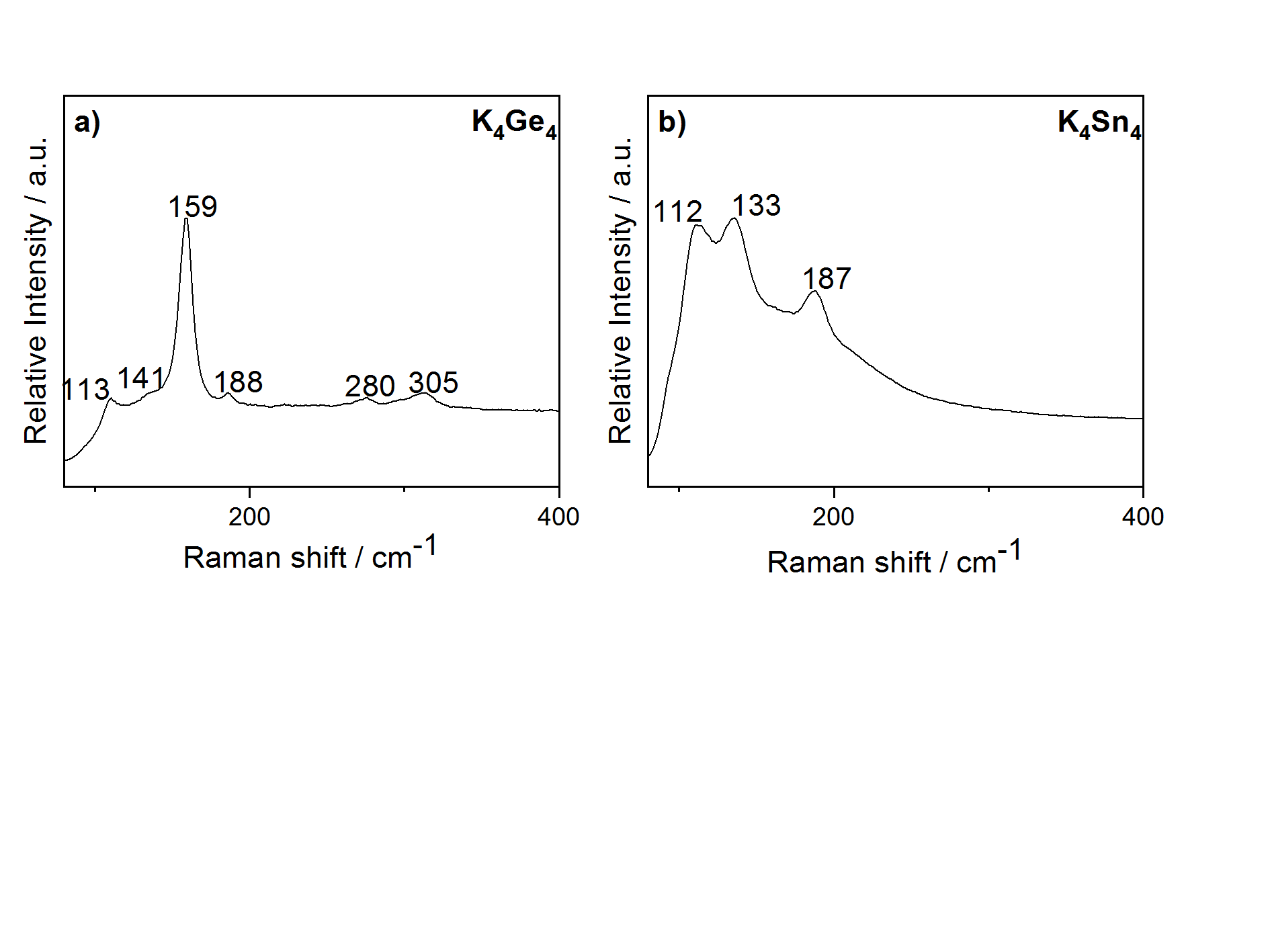


**Figure S16:** Raman spectra of *Zintl* clusters with the nominal composition a) K_4_Ge_4_ and b) K_4_Sn_4_. The vibration frequencies of the experimental and literature known bands are in good agreement (lit. for K_4_Sn_4_: 85, 100, 118, 133, 183 cm^−1^ and lit. for K_4_Ge_4_: 83, 106, 135, 158, 185, 200, 274 cm^−1^).^[6]^ The detection of the band at 85 cm^-1^ for K_4_Sn_4_ and the band at 83 cm^−1^ for K_4_Ge_4_ is not possible due to the Rayleigh filter cutting of signals below 110 cm^−1^.

5.4. PXRD and FTIR of LiCl


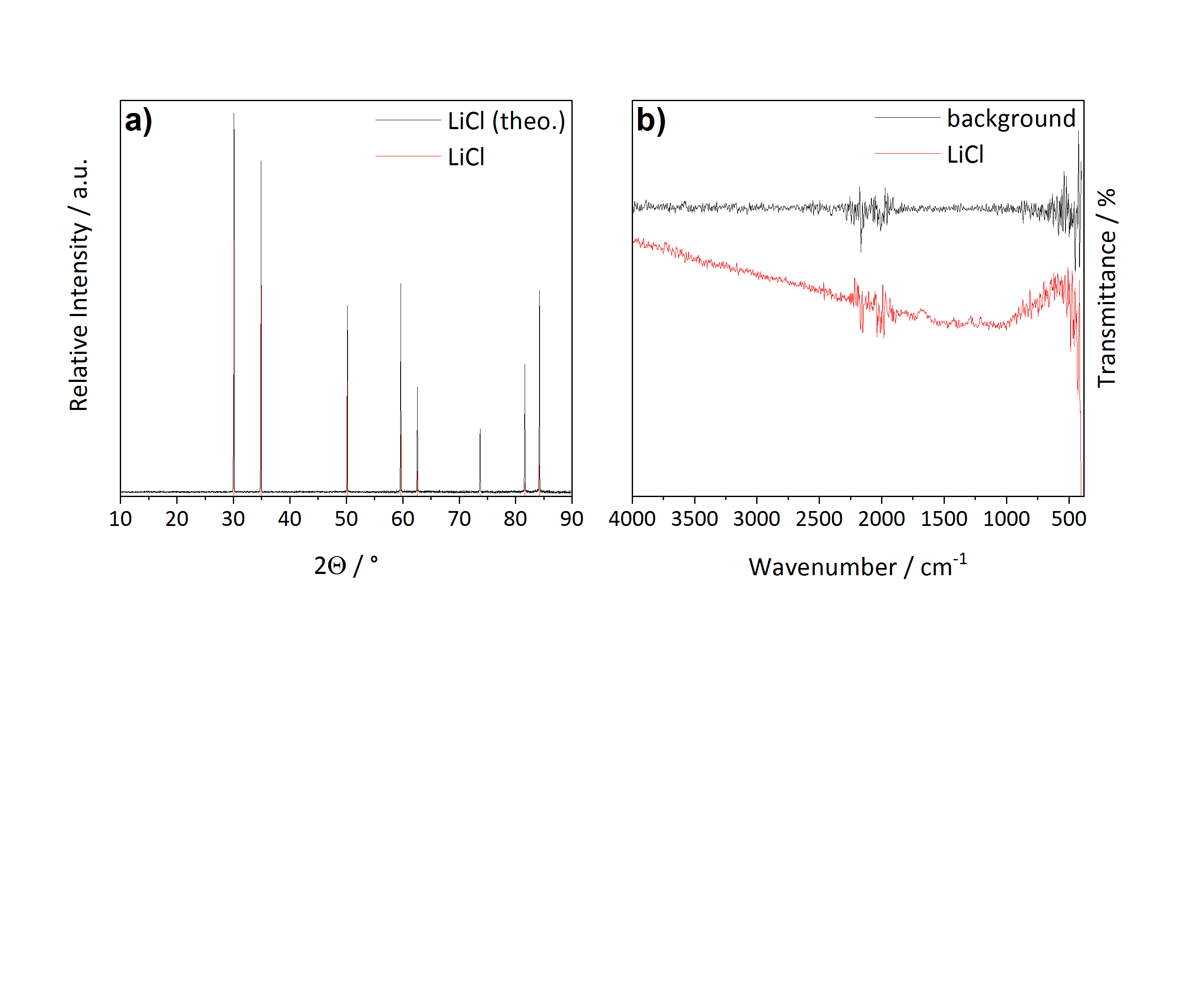


**Figure S17:** a) Powder X-ray diffraction of LiCl. All reflections can be assigned to the theoretical pattern calculated from single crystal data indicating that no other crystalline compound such as LiCl*H_2_O is present in the sample. b) FTIR spectra of a blank measurement and LiCl. The absence of any significant band between 3200 and 3600 cm^−1^ confirms that LiCl which is used in this work is completely anhydrous. The noisy background between 2000 and 2500 cm^−1^ is an artifact of the spectrometer, as shown by comparison of both measurements.

5.5. PXRD of the Precipitate


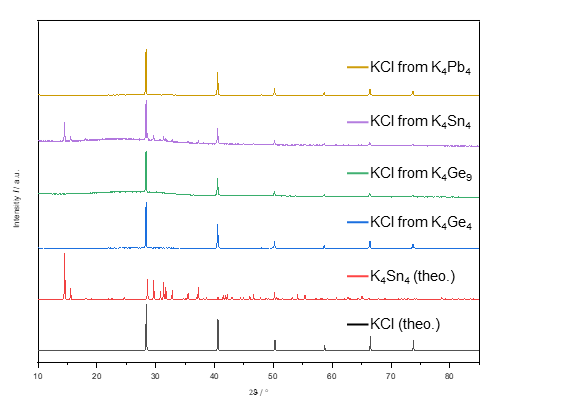


**Figure S18:** Powder X-ray diffraction of the residual solid obtained from the reaction solutions of the respective *Zintl* phases with LiCl. The solid was isolated from the solution by filtration and dried under vacuum.

6. Crystallographic Data

**Table S4**: Crystallographic data of compounds **1**, **2**, and **3**.

| Compound | [Li(*en*)_2.5_]_4_[Ge_9_] (**1**) | [Li(*en*)_2_]_4_[Sn_9_] (**2**) | [Li(*en*)_2_]_4_[Pb_9_] (**3**) |
| --- | --- | --- | --- |
| formula | Li_4_C_20_N_20_H_80_Ge_9_ | Li_4_C_16_N_16_H_64_Sn_9_ | Li_4_C_16_N_16_H_64_Pb_9_ |
| fw [g·mol^−1^] | 1282.11 | 1576.80 | 2373.30 |
| space group | *P*2_1_/*c* (no. 14) | *P*$\overline{1}$ (no. 2) | *Pbcn* (no. 60) |
| *a* [Å] | 16.8306(4) | 12.2977(5) | 14.2732(8) |
| *b* [Å] | 11.2802(2) | 12.5647(5) | 17.4079(6) |
| *c* [Å] | 27.2191(7) | 17.7472(6) | 18.9145(6) |
| *α* [°] | 90 | 74.131(3) | 90 |
| *β* [°] | 106.099(2) | 71.143(3) | 90 |
| *γ* [°] | 90 | 62.655(3) | 90 |
| *V* [Å^3^] | 4965.0(2) | 2279.46(17) | 4699.6(3) |
| Z | 4 | 2 | 4 |
| *T* [K] | 150(2) | 150(2) | 150(2) |
| *λ* [Å] | 0.71073 | 0.71073 | 0.71073 |
| *ρ*_calcd_ [g·cm^−3^] | 1.715 | 2.297 | 3.354 |
| *μ* [mm^−1^] | 5.406 | 4.873 | 32.143 |
| collected reflections | 128032 | 51063 | 109989 |
| *R*_int_ | 0.0369 | 0.0307* | 0.1039 |
| independent reflections | 9739 | 51063 | 4627 |
| reflections > 2 *σ*(*I*) | 8275 | 39052 | 3521 |
| parameters / restraints | 570 / 12 | 407 / 0 | 204 / 0 |
| *R_1_* [*I* > 2 *σ*(*I*) / all data] | 0.0260 / 0.0361 | 0.0295 / 0.0471 | 0.0280 / 0.0478 |
| w*R_2_* [*I* > 2 *σ*(*I*) / all data] | 0.0555 / 0.0604 | 0.0673 / 0.0765 | 0.0508 / 0.0548 |
| goodness of fit | 1.060 | 1.095 | 0.992 |
| max./min. diff. el. density [e/Å^−3^] | 1.144 / −1.021 | 1.738 / −1.608 | 1.965 / −2.392 |
| depository no. | CCDC-2289462 | CCDC-2289463 | CCDC-2289464 |

* for HKLF4 data

**Table S5**. Crystallographic data of compounds **4** and **5**.

| Compound | [Li_6_(*en*)_13_]Ge_18_ (**4**) | [Li(*en*)]_4_K_2_[Ge_18_] (**5**) |
| --- | --- | --- |
| formula | C_13_H_52_Ge_9_Li_3_N_13_ | C_8_H_32_Ge_9_KLi_2_N_8_ |
| fw [g·mol^−1^] | 1064.80 | 946.70 |
| space group | *P*2_1_/*n* (no. 14) | *P*2_1_/*n* (no. 14) |
| *a* [Å] | 12.6835(7) | 9.3575(5) |
| b [Å] | 19.9429(13) | 15.2339(7) |
| *c* [Å] | 14.8428(8) | 19.0962(8) |
| *α* [°] | 90 | 90 |
| *β* [°] | 101.854(4) | 94.528(4) |
| *γ* [°] | 90 | 90 |
| *V* [Å^3^] | 3674.4(4) | 2713.7(2) |
| Z | 4 | 4 |
| *T* [K] | 150(2) | 150(2) |
| *λ* [Å] | 0.71073 | 0.71073 |
| *ρ*_calcd_ [g·cm^−3^] | 1.925 | 2.317 |
| *μ* [mm^−1^] | 7.279 | 9.985 |
| collected reflections | 71794 | 52291 |
| *R*_int_ | 0.0584 | 0.0823 |
| independent reflections | 7219 | 5335 |
| reflections > 2 *σ*(*I*) | 5366 | 3974 |
| parameters / restraints | 453 / 0 | 253 / 0 |
| *R_1_* [*I* > 2 *σ*(*I*) / all data] | 0.0513 / 0.0796 | 0.0398 / 0.0694 |
| w*R_2_* [*I* > 2 *σ*(*I*) / all data] | 0.1120 / 0.1267 | 0.0720 / 0.0809 |
| goodness of fit | 1.053 | 1.059 |
| max./min. diff. el. density [e/Å^−3^] | 2.545 / −0.982 | 1.191 / −0.753 |
| depository no. | CCDC-2289465 | CCDC-2289466 |

**Table S6**. Prism heights *h*_1_, *h*_2_ and *h*_3_ of the trigonal prism are normalized to the shortest prism height of anions **1** to **5** in bold and as a reference literature known anions are shown in regular font. The ratio of the diagonals of the quadratic plane are given for a quadratic anti prism. For anions with positional disorder **1** and **4** the values are given for both individuals, [a] and [c] represent the higher, [b] and [d] the lower occupied cluster. In compounds with two cryptographically independent cluster anions the anions are individually analysed and displayed as I and II.

|  | ***h*_1_** | ***h*_2_** | ***h*_3_** | ***d*_1_/*d*_2_** | **point group** |
| --- | --- | --- | --- | --- | --- |
| **[Ge_9_]^4-^ (1)^[a]^** | 1 | 1.08 | 1.10 | 1.27 | *C*_2_*_v_* |
| **[Ge_9_]^4-^ (1)**^[b]^ | 1 | 1.04 | 1.15 | 1.14 | *C*_2_*_v_* |
| [K(18-crown-6)]_2_K_2_[Ge_9_] | 1 | 1.037 | 1.055 | 1.258 | *D_3h_* |
| Rb_4_Ge_9_ · *en* (I)^[7]^ | 1 | 1.026 | 1.146 | 1.139 | *C*_2_*_v_* |
| Rb_4_Ge_9_ · *en* (II)^[7]^ | 1 | 1.006 | 1.285 | 1 | *C*_4_*_v_* |
| Cs_4_Ge_9_ · *en* (I)^[8]^ | 1 | 1.021 | 1.299 | 1.019 | *C*_4_*_v_* |
| Cs_4_Ge_9_ · *en* (II)^[8]^ | 1 | 1.053 | 1.237 | 1.093 | *C*_4_*_v_* |
| **[Sn_9_]^4-^ (2)** | 1 | 1.01 | 1.24 | 1.08 | *C*_4_*_v_* |
| [Na(2.2.2-crypt)]_4_Sn_9_^[9]^ | 1 | 1.01 | 1.31 | 1.01 | *C*_4_*_v_* |
| [K(18-crown-6)]_2_K_2_[Sn_9_]^[10]^ | 1 | 1.00 | 1.29 | 1.02 | *C*_4_*_v_* |
| [K(12-crown-4)_2_]_2_[K(12-crown-4)]_2_[Sn_9_] · 4 *en*^[11]^ | 1 | 1.00 | 1.09 | 1.22 | *D_3h_* |
| [Rb(2.2.2-crypt)]_3_Rb[Sn_9_]^[12]^ | 1 | 1.03 | 1.27 | 1.03 | *C*_4_*_v_* |
| [K(2.2.2-crypt)]Cs_7_[Sn_9_]_2_ · 3 *en*^[13]^ | 1 | 1.01 | 1.30 | 1.01 | *C*_4_*_v_* |
| **[Pb_9_]^4-^ (3)** | 1 | 1.09 | 1.09 | 1.24 | *D_3h_* |
| [K(18-crown-6)]_4_[Pb_9_] · *en* · tol^[14]^ | 1 | 1.04 | 1.33 | 1.00 | *C*_4_*_v_* |
| [K(18-crown-6)]_2_K_2_[Pb_9_] · 1.5 *en*^[15]^ | 1 | 1.01 | 1.29 | 1.01 | *C*_4_*_v_* |
| [K(2.2.2-crypt)]_3_K[Pb_9_]^[16]^ | 1 | 1.02 | 1.29 | 1.01 | *C*_4_*_v_* |
| **[Ge_18_]^6-^ (4)^[c]^** | 1 | 1.13 | 1.16 | 1.07 | *C*_4_*_v_* |
| **[Ge_18_]^6-^ (4)^[d]^** | 1 | 1.03 | 1.17 | 1.03 | *C*_4_*_v_* |
| **[Ge_18_]^6-^ (5)** | 1 | 1.01 | 1.34 | 1.08 | *C*_4_*_v_* |
| K_4_[K(2.2.2-crypt)]_2_[Ge_9_−Ge_9_] ⋅ 4 *en* ⋅ 2 thf^[17]^ | 1 | 1.011 | 1.242 | 1.134 | *C*_2_*_v_* |
| K_4_[K(2.2.2-crypt)]_2_[Ge_9_−Ge_9_] ⋅ 4 *en*^[18]^ | 1 | 1.012 | 1.192 | 1.167 | *C*_2_*_v_* |
| [K(C_18_H_36_N_2_O_6_)]_2_K_4_[Ge_9_−Ge_9_] · 6 *en*^[19]^ | 1 | 1.011 | 1.195 | 1.16 | *C*_2_*_v_* |
| Rb_4_[Rb(2.2.2-crypt)]_2_[Ge_9_−Ge_9_] ⋅ 7 *en*^[18]^ | 1 | 1.008 | 1.311 | 1.033 | *C*_4_*_v_* |
| [Rb_6_(Ge_9_−Ge_9_)] ⋅ 12 dmf^[20]^ | 1 | 1.008 | 1.227 | 1.133 | *C*_2_*_v_* |
| [K2.2.2-crypt]_4_[Mg(NacNac^Mes^)(NH_3_)_4_]_2_[Ge_9_−Ge_9_] ⋅ 32.58 NH_3_^[21]^ | 1 | 1.01 | 1.27 | 1.09 | *C*_4_*_v_* |

Crystal packing is affecting the space group and crystal shape of **1** – **3**. Such a behavior has been previously observed in the rubidium and cesium solvates of [*E*_9_]^4-^, which crystalize as the same *en* solvate (*A*_4_Ge_9_ · *en, A= Rb or Cs*) but are not isostructural.^[7, 8]^


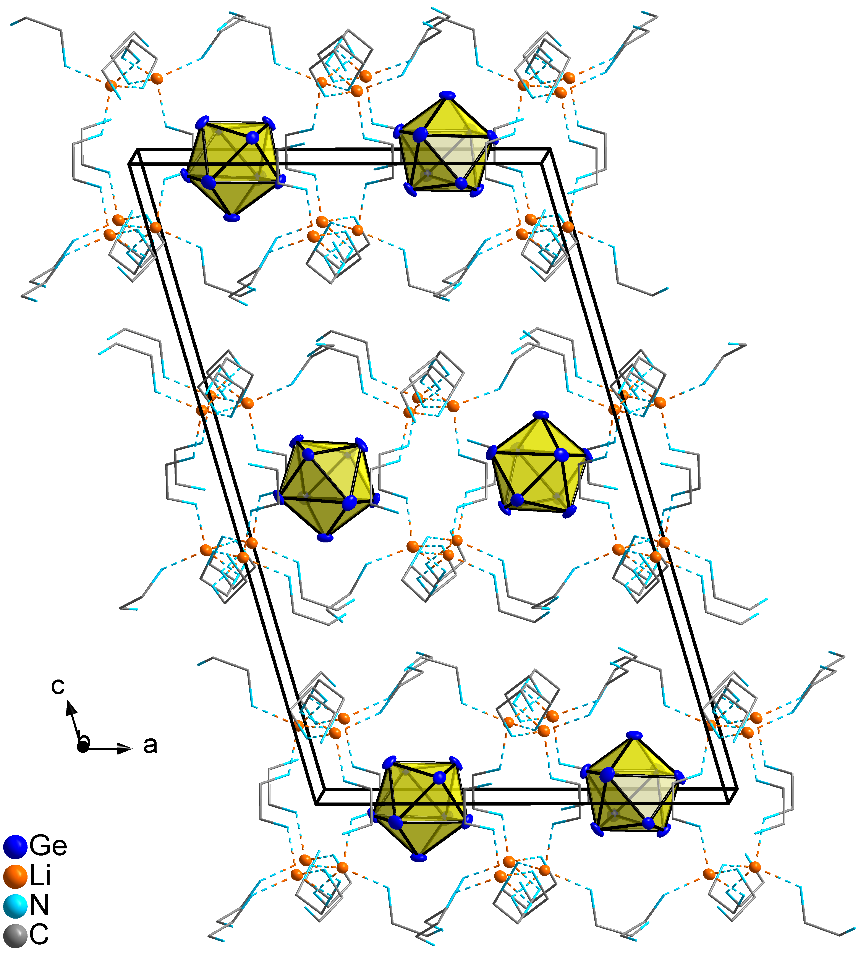


**Figure S19**: View of the [Li_4_(en)_10_]^4+^ network that embeds the anion in compound **1**. Nitrogen and carbon atoms are shown in wire and stick mode. Positions of disordered atoms and hydrogen atoms have been omitted for clarity.


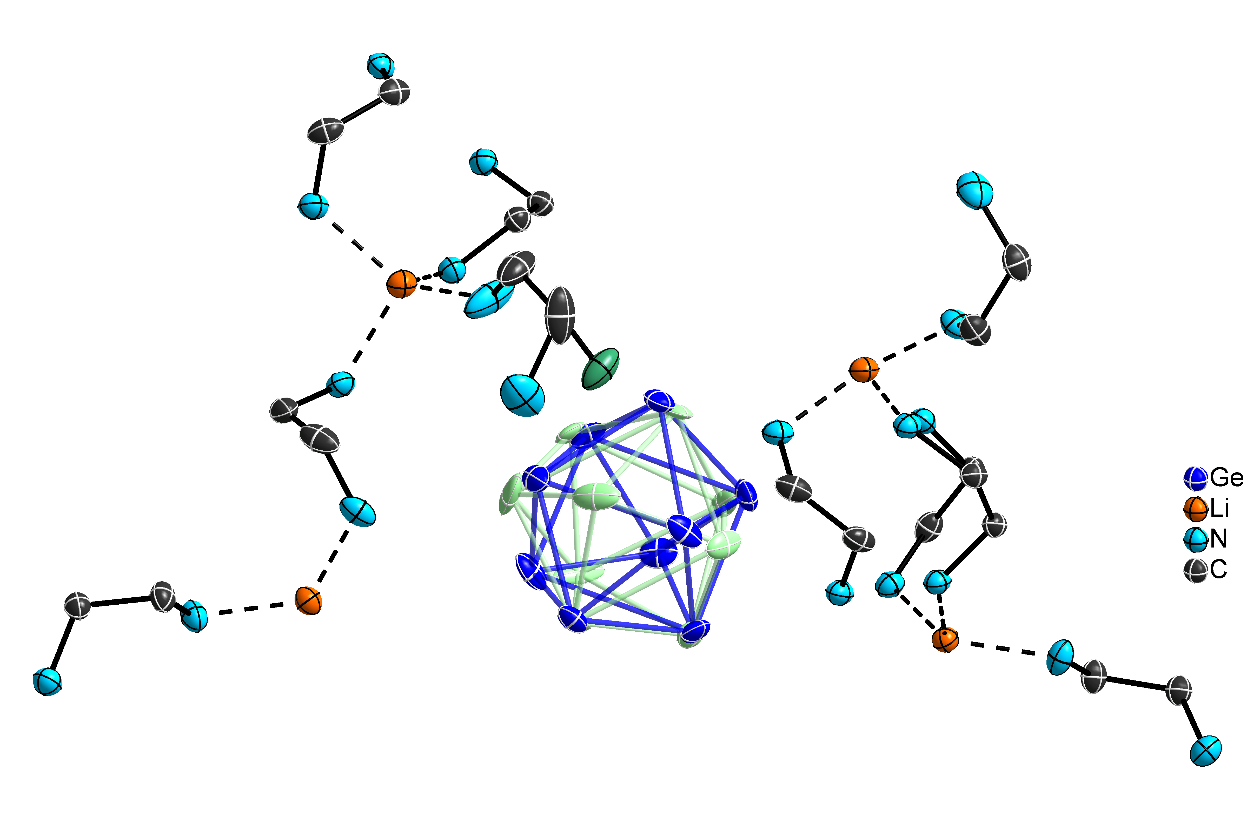


**Figure S20:** The asymmetric unit of compound **1** with the cluster. Thermal ellipsoids are drawn at 50% probability. Positions of disordered atoms are shown semi-transparent. The disordered germanium atoms are shown in blue and light green. The disordered nitrogen atom is shown in dark green. Hydrogen atoms are omitted for clarity.


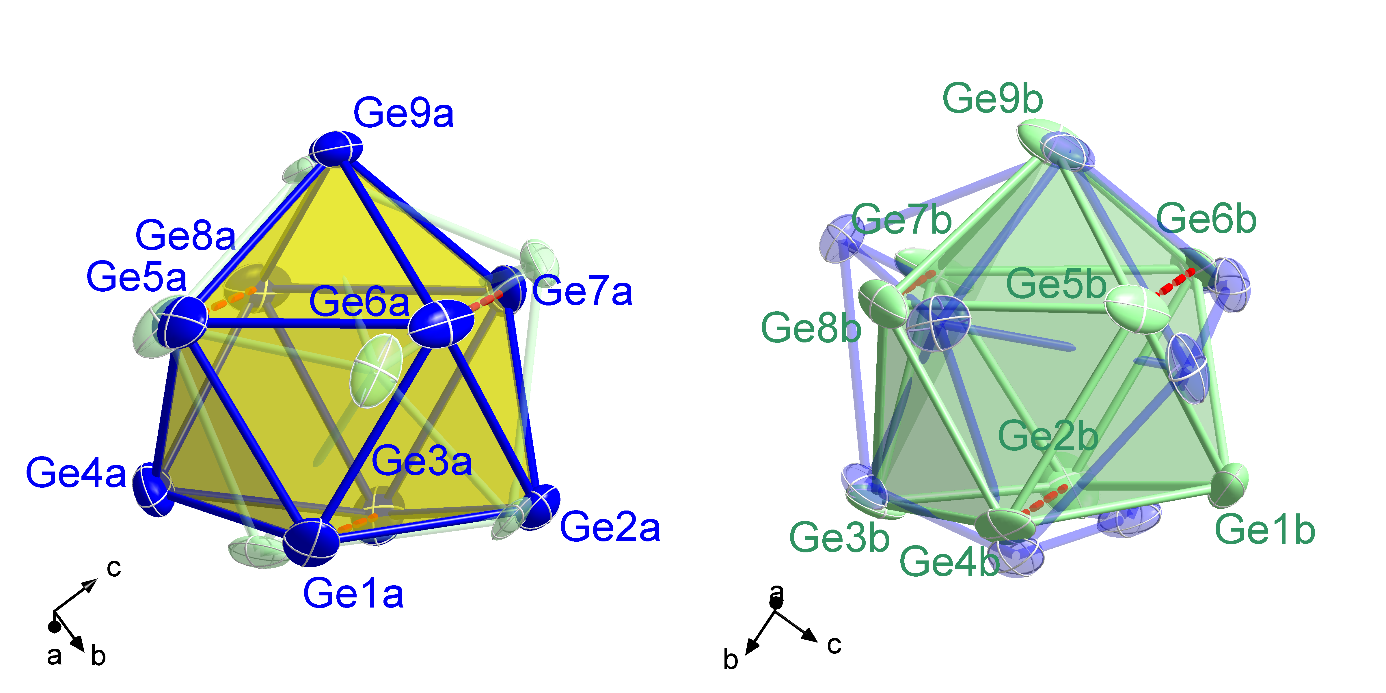


**Figure S21:** The two individuals of the positionally disordered Ge_9_ cluster in compound **1**, left: main individual, s.o.f. 90.08(8)%; right: minor individual, s.o.f. 9.92(8)%. In each case, the atoms of the other individual are drawn in semi-transparent manner. Thermal ellipsoids are drawn at 50% probability.


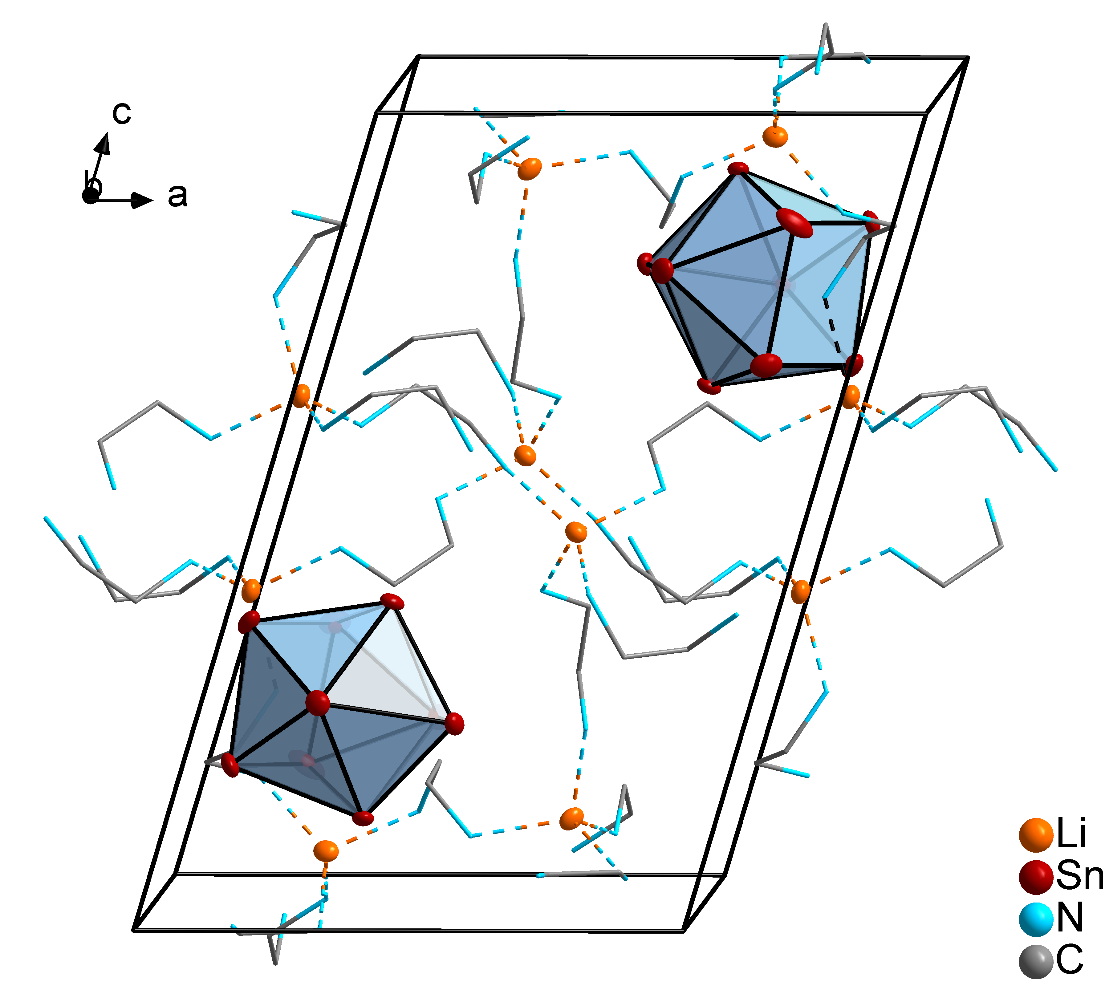


**Figure S22**: Extended unit cell of compound **2**. Nitrogen and carbon atoms are shown in wire and stick mode. Hydrogen atoms have been omitted for clarity.


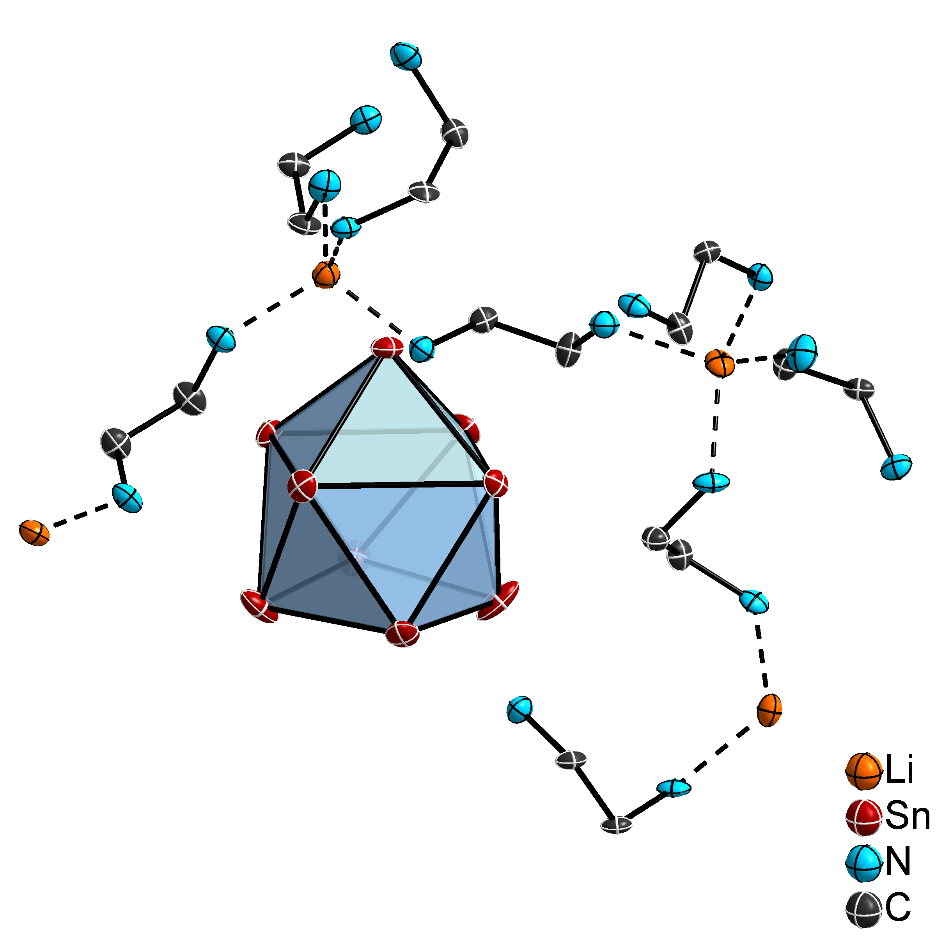


**Figure S23**: The asymmetric unit of compound **2** with the cluster. Thermal ellipsoids are drawn at 50% probability. Hydrogen atoms are omitted for clarity.


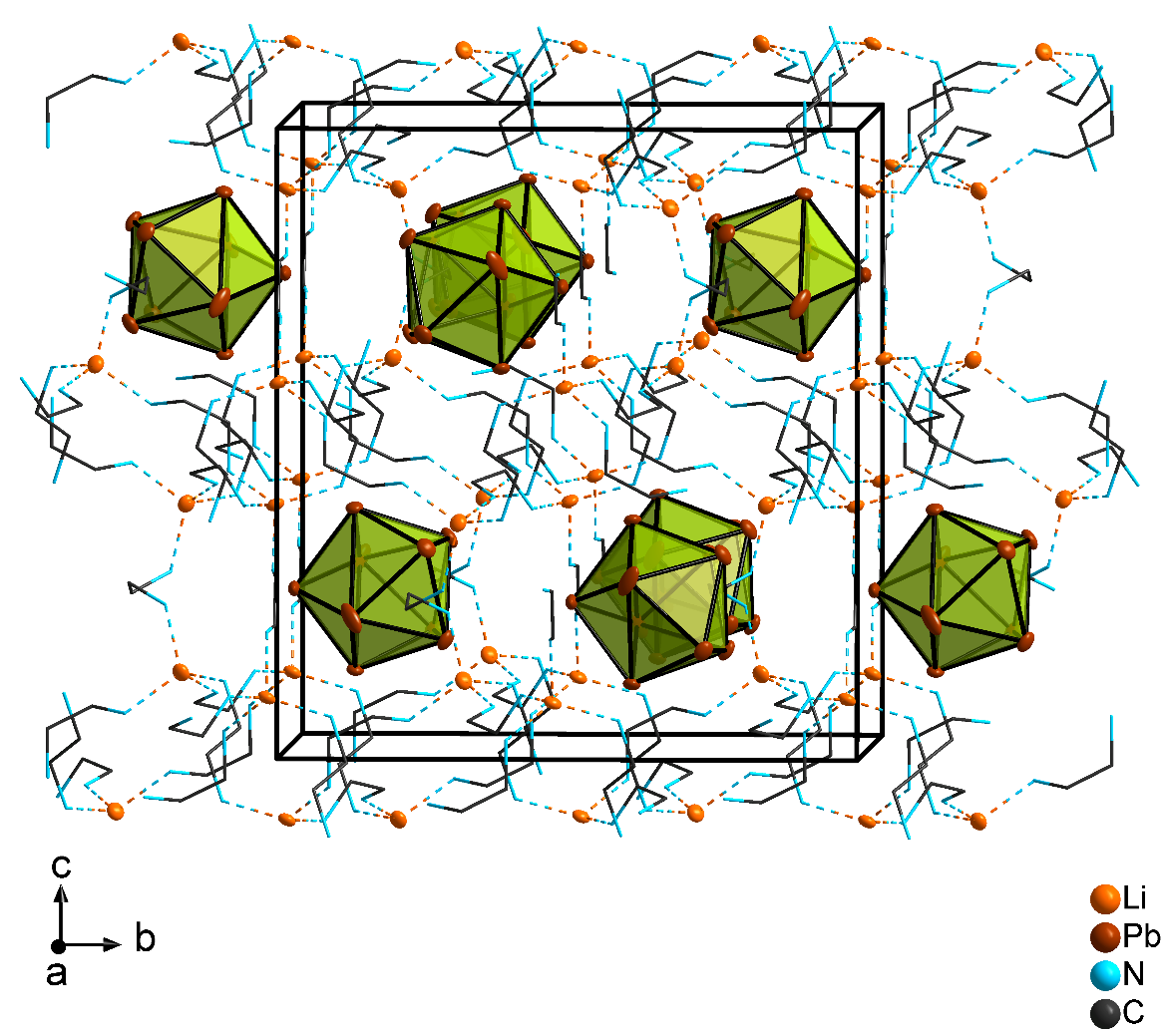


**Figure S24**: Extended unit cell of compound **3**. Nitrogen and carbon atoms are shown in wire and stick mode. Hydrogen atoms have been omitted for clarity.


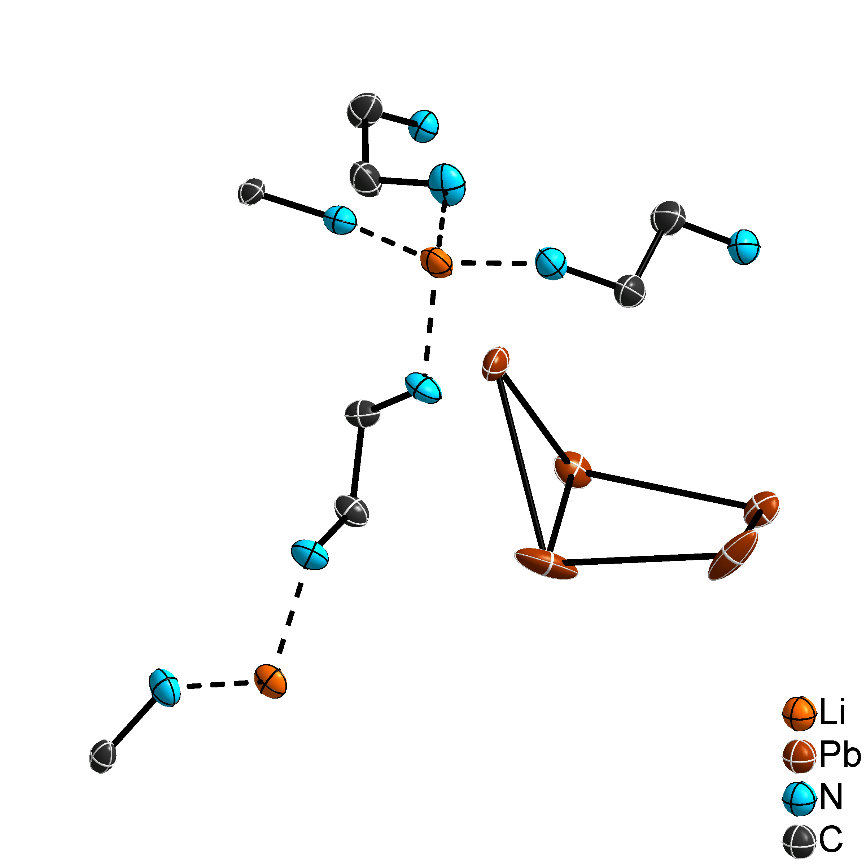


**Figure S25**: The asymmetric unit of compound **3** with the cluster fragment. Thermal ellipsoids are drawn at 50% probability. Hydrogen atoms are omitted for clarity.


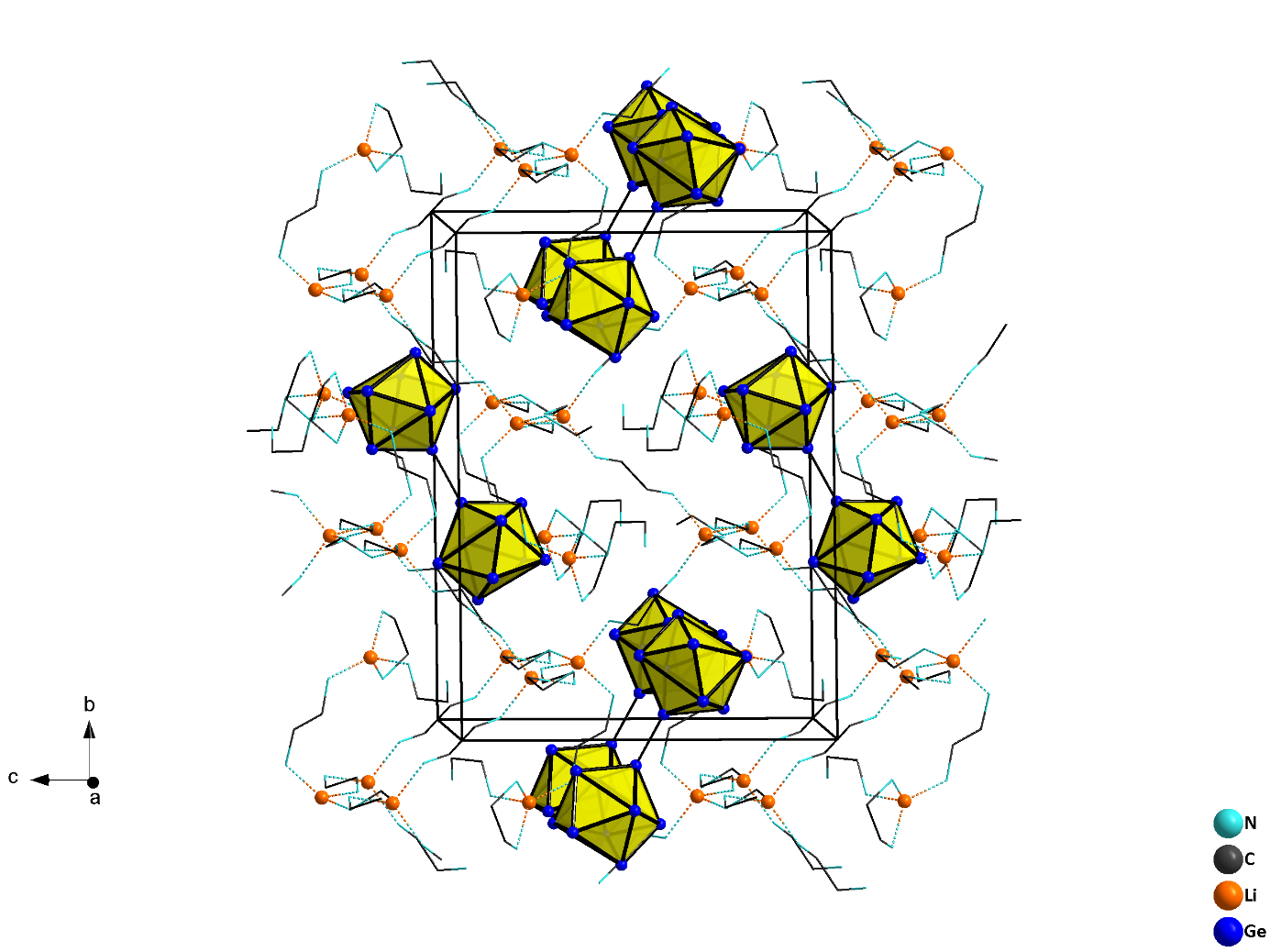


**Figure S26**: Extended unit cell of compound **4**. Nitrogen and carbon atoms are shown in wire and stick mode. Hydrogen atoms have been omitted for clarity.


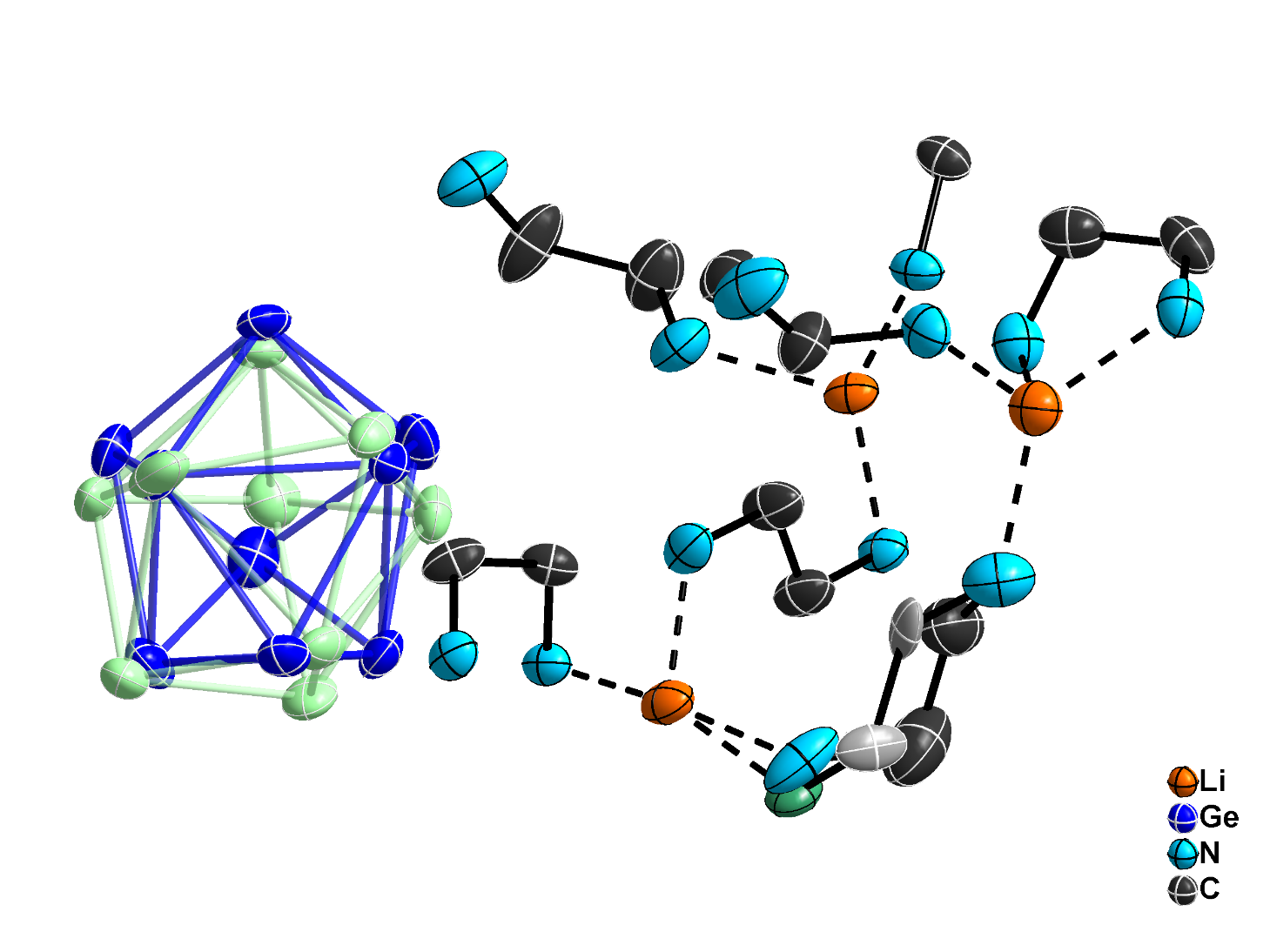


**Li2**

**Li1**

**Li3**

**Figure S27:** The asymmetric unit of compound **4** with the cluster. Thermal ellipsoids are drawn at 50% probability. Positions of disordered germanium atoms are shown semi-transparent manner. The disordered germanium atoms are shown in blue and light green. The disordered nitrogen atom is shown in dark green and the disordered carbon atoms are shown in light grey. Hydrogen atoms are omitted for clarity.

In compound **4**, an odd ratio of Li^+^ ions to *en* (of 6 to 13) is found due to the presence of a “dangling” *en* molecule in the cation/solvent network. In total three different coordination modes for the *en* molecule coordinated to Li3 are found. One *en* molecule coordinates in a bridging manner between Li1 and Li3, the second one in a dangling fashion, where one amine group is not coordinating to a second Li^+^ ion, while the third kind of *en* molecules coordinates with both amine groups to the same Li^+^ ion as chelating ligand (see Figure S27). Therefore, the network of alternating cations and solvent molecules terminates at the Li3 position and forms an “end group”.


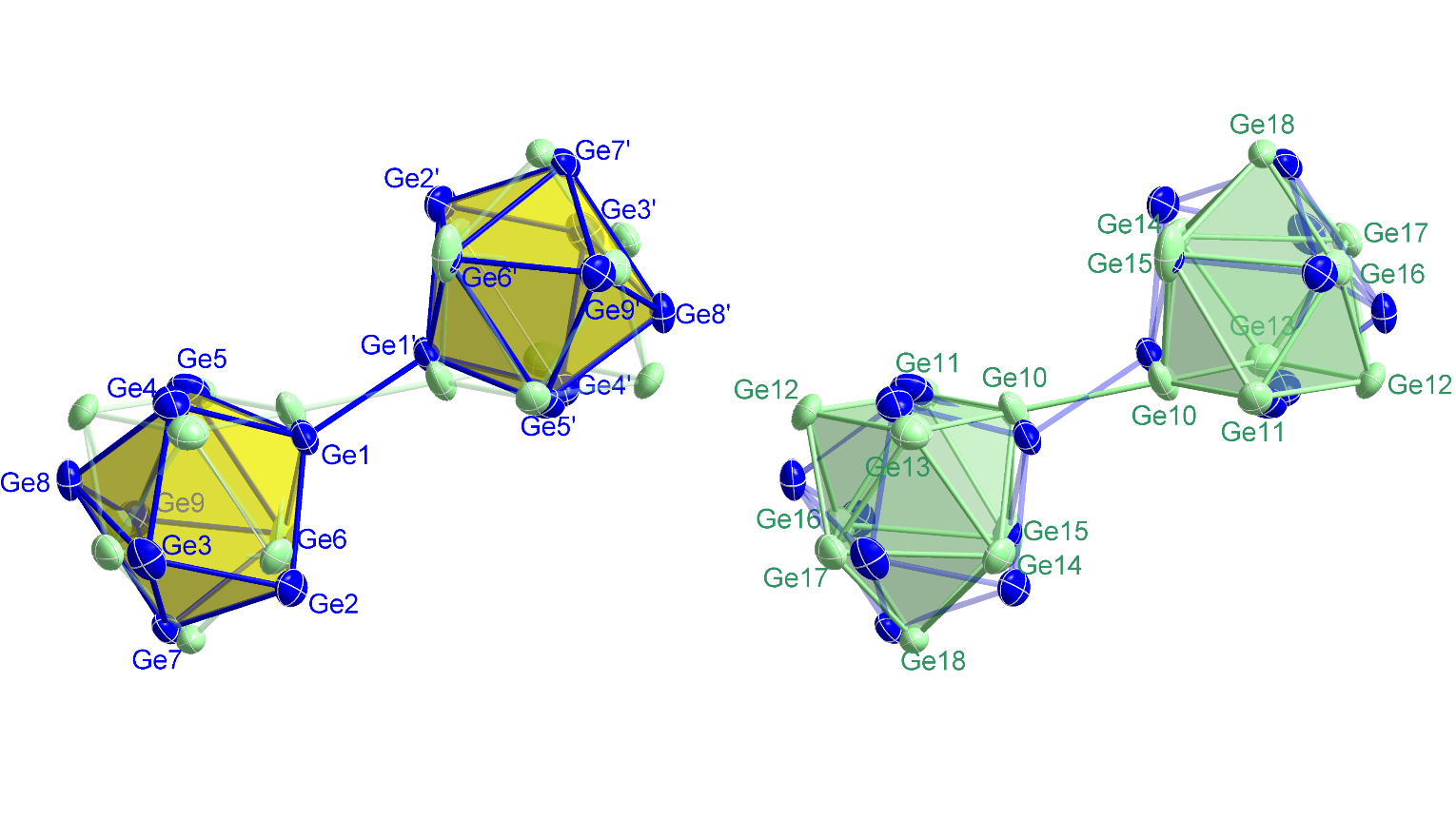


**Figure S28:** The two individuals of the positionally disordered Ge_9_-Ge_9_ cluster in compound **5**, left: main individual, s.o.f. 61.3(2) %; right: minor individual, s.o.f. 38.7(2) %. In each case, the atoms of the other individual are drawn in semi-transparent manner. Thermal ellipsoids are drawn at 50% probability.

In compound **4,** the cluster shows positional disorder over all atom positions with occupations of 61.3(2) % to 38.7(2) %% (Figure S28). The disorder can be described as a rotation of the anionic cluster by approximately 90°.


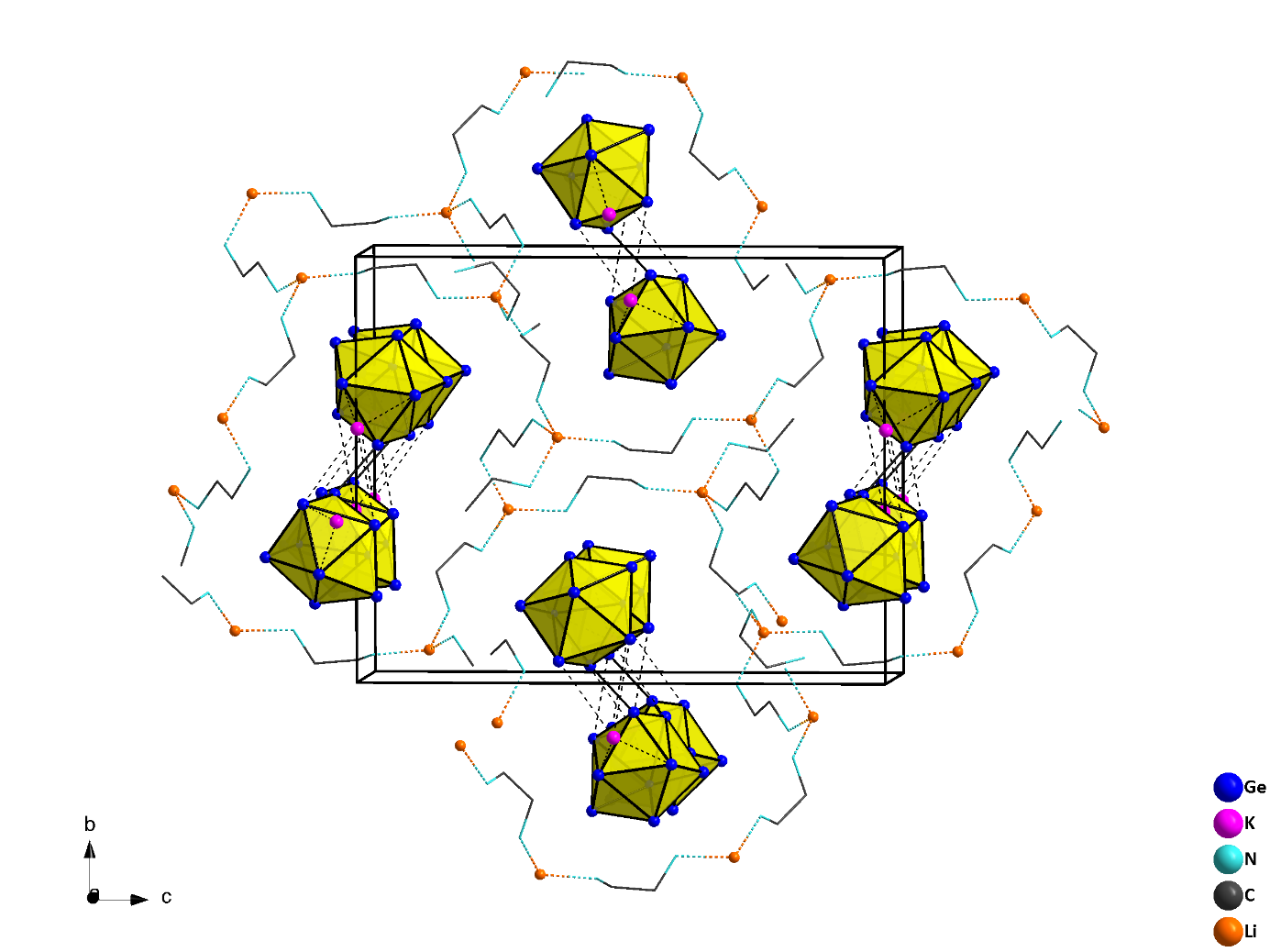


**Figure S29**: Extended unit cell of compound **5**. Nitrogen and carbon atoms are shown in wire and stick mode. Hydrogen atoms have been omitted for clarity.


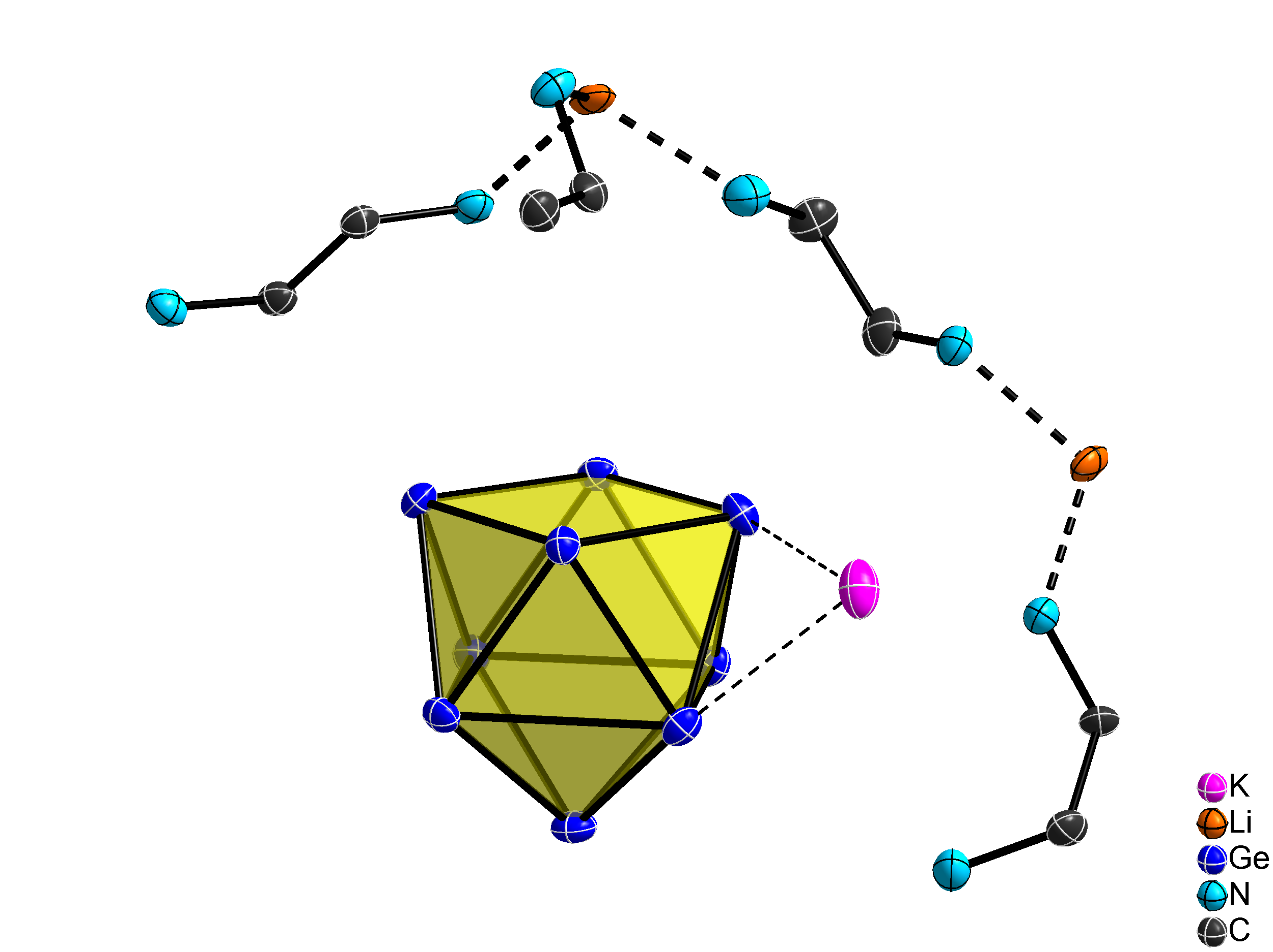


**Figure S30**: The asymmetric unit of compound **5** with the cluster. Thermal ellipsoids are drawn at 50% probability. Hydrogen atoms are omitted for clarity.

**Table S7**: Selected interatomic distances in **1**.

| Atoms | Distance [Å] | Atoms | Distance [Å] |
| --- | --- | --- | --- |
| Ge1a-Ge2a | 2.5631(13) | Ge1b-Ge2b | 2.571(6) |
| Ge2a-Ge3a | 2.572(3) | Ge2b-Ge3b | 2.13(2) |
| Ge3a-Ge4a | 2.555(2) | Ge3b-Ge4b | 2.74(3) |
| Ge4a-Ge1a | 2.5757(6) | Ge4b-Ge1b | 2.614(17) |
| Ge1a-Ge5a | 2.605(3) | Ge1b-Ge5b | 2.551(6) |
| Ge1a-Ge6a | 2.6247(6) | Ge1b-Ge6b | 2.609(12) |
| Ge2a-Ge6a | 2.5873(19) | Ge2b-Ge6b | 2.631(15) |
| Ge2a-Ge7a | 2.5826(18) | Ge2b-Ge7b | 2.575(15) |
| Ge3a-Ge7a | 2.711(3) | Ge3b-Ge7b | 2.50(2) |
| Ge3a-Ge8a | 2.630(3) | Ge3b-Ge8b | 2.97(2) |
| Ge4a-Ge8a | 2.5759(6) | Ge4b-Ge8b | 2.587(14) |
| Ge4a-Ge5a | 2.575(3) | Ge4b-Ge5b | 2.603(16) |
| Ge5a-Ge6a | 2.697(3) | Ge5b-Ge6b | 2.998(15) |
| Ge6a-Ge7a | 2.8946(7) | Ge6b-Ge7b | 2.691(10) |
| Ge7a-Ge8a | 2.6228(6) | Ge7b-Ge8b | 2.891(7) |
| Ge8a-Ge5a | 3.171(8) | Ge8b-Ge5b | 2.633(6) |
| Ge5a-Ge9a | 2.597(4) | Ge5b-Ge9b | 2.39(3) |
| Ge6a-Ge9a | 2.5770(16) | Ge6b-Ge9b | 2.41(3) |
| Ge7a-Ge9a | 2.5775(17) | Ge7b-Ge9b | 2.59(3) |
| Ge8a-Ge9a | 2.5637(12) | Ge8b-Ge9b | 2.54(3) |
| Ge1a-Ge3a | 3.139(2) | Ge1b-Ge3b | 3.77(2) |
| Ge2a-Ge4a | 4.0134(17) | Ge2b-Ge4b | 3.309(17) |
|  |  |  |  |
| Li1-N1 | 2.063(5) | Li3-N17 | 2.067(5) |
| Li1-N13 | 2.064(5) | Li3-N9^i^ | 2.070(5) |
| Li1-N5^i^ | 2.070(4) | Li3-N11^i^ | 2.079(5) |
| Li1-N3 | 2.093(4) | Li3-N7 | 2.096(5) |
| Li2-N2 | 2.067(5) | Li4-N10 | 2.055(5) |
| Li2-N15 | 2.076(5) | Li4-N19 | 2.062(5) |
| Li2-N4^i^ | 2.093(5) | Li4-N8 | 2.092(5) |
| Li2-N6 | 2.105(5) | Li4-N12 | 2.095(5) |

**Table S8**: Possible hydrogen bonds in **1**, distances in Å.

| **Atoms** | **N-H distances** | **H∙∙∙Ge distances** | **N∙∙∙Ge distances** | **N-H∙∙∙Ge angles** |
| --- | --- | --- | --- | --- |
| N6-H6d∙∙∙Ge6a | 0.91 | 2.74 | 3.644(2) | 176.0° |
| N7-H7d∙∙∙Ge7a | 0.91 | 2.81 | 3.713(2) | 174.0° |
| N11-H11c∙∙∙Ge8a^i^ | 0.91 | 2.84 | 3.681(2) | 154.3° |
| N4-H4d∙∙∙Ge1a | 0.91 | 2.85 | 3.689(2) | 154.4° |
| N3-H3d∙∙∙Ge1a | 0.91 | 2.89 | 3.758(2) | 160.3° |
| N11-H11d∙∙∙Ge9a^ii^ | 0.91 | 2.90 | 3.792(3) | 168.2° |

**Table S9**: Selected interatomic distances in **2**.

| Atoms | Distance [Å] | Atoms | Distance [Å] |
| --- | --- | --- | --- |
| Sn1-Sn2 | 2.9665(8) | Li1-N4 | 2.029(13) |
| Sn2-Sn3 | 2.9773(7) | Li1-N1 | 2.059(12) |
| Sn3-Sn4 | 2.9703(8) | Li1-N6 | 2.086(12) |
| Sn4-Sn1 | 2.9568(7) | Li1-N16 | 2.132(11) |
| Sn1-Sn5 | 2.9817(7) | Li2-N7 | 2.034(11) |
| Sn1-Sn6 | 2.9393(7) | Li2-N2 | 2.049(11) |
| Sn2-Sn6 | 2.9694(7) | Li2-N3 | 2.097(13) |
| Sn2-Sn7 | 2.9698(8) | Li2-N5 | 2.109(13) |
| Sn3-Sn7 | 2.9483(7) | Li3-N8 | 2.056(11) |
| Sn3-Sn8 | 2.9488(7) | Li3-N9 | 2.071(12) |
| Sn4-Sn8 | 2.9841(7) | Li3-N11 | 2.078(12) |
| Sn4-Sn5 | 2.9770(7) | Li3-N13 | 2.104(13) |
| Sn5-Sn6 | 3.2460(6) | Li4-N10 | 2.050(12) |
| Sn6-Sn7 | 3.1429(7) | Li4-N14 | 2.076(13) |
| Sn7-Sn8 | 3.2722(6) | Li4-N12 | 2.086(11) |
| Sn8-Sn5 | 3.1125(7) | Li4-N15 | 2.108(12) |
| Sn5-Sn9 | 2.9560(7) |  |  |
| Sn6-Sn9 | 2.9449(6) |  |  |
| Sn7-Sn9 | 2.9798(7) |  |  |
| Sn8-Sn9 | 2.9515(6) |  |  |
| Sn1-Sn3 | 4.3606(7) |  |  |
| Sn2-Sn4 | 4.0200(7) |  |  |

**Table S10**: Shortest possible hydrogen bonds in **2**, distances in Å.

| **Atoms** | **N-H distances** | **H∙∙∙Sn distances** | **N∙∙∙Sn distances** | **N-H∙∙∙Sn angles** |
| --- | --- | --- | --- | --- |
| N9-H9c∙∙∙Sn2^i^ | 0.91 | 3.00 | 3.887(5) | 163.7° |
| N6-H6d∙∙∙Sn4^ii^ | 0.91 | 3.01 | 3.859(5) | 155.9° |
| N12-H12d∙∙∙Sn6^i^ | 0.91 | 3.05 | 3.928(6) | 162.7° |
| N10-H10c∙∙∙Sn6 | 0.91 | 3.05 | 3.921(5) | 160.6° |
| N6-H6c∙∙∙Sn3^iii^ | 0.91 | 3.07 | 3.861(5) | 146.6° |
| N15-H15c∙∙∙Sn5^iv^ | 0.91 | 3.08 | 3.869(6) | 145.8° |
| N14-H14c∙∙∙Sn6^i^ | 0.91 | 3.08 | 3.943(5) | 158.4° |
| N7-H7c∙∙∙Sn7 | 0.91 | 3.09 | 3.940(6) | 156.2° |
| N3-H3c∙∙∙Sn8^v^ | 0.91 | 3.10 | 3.932(5) | 152.1° |

**Table S11**: Selected interatomic distances in **3**.

| Atoms | Distance [Å] | Atoms | Distance [Å] |
| --- | --- | --- | --- |
| Pb1-Pb2 | 3.5065(4) | Li1-N1 | 2.023(16) |
| Pb2-Pb3 | 3.0973(5) | Li1-N3 | 2.085(14) |
| Pb3-Pb4 | 3.0823(5) | Li1-N5 | 2.074(16) |
| Pb4-Pb1 | 3.0968(5) | Li1-N7 | 2.090(15) |
| Pb1-Pb2^i^ | 3.5065(4) | Li2-N2 | 2.038(15) |
| Pb1-Pb4^i^ | 3.0969(5) | Li2-N4 | 2.097(14) |
| Pb2-Pb4^i^ | 3.1214(5) | Li2-N6 | 2.059(15) |
| Pb2-Pb5^i^ | 3.1834(5) | Li2-N8 | 2.054(14) |
| Pb3-Pb5^i^ | 3.0903(5) |  |  |
| Pb3-Pb5 | 3.0738(5) |  |  |
| Pb4-Pb5 | 3.1844(5) |  |  |
| Pb4-Pb2^i^ | 3.1214(5) |  |  |
| Pb4^i^-Pb5^i^ | 3.1844(5) |  |  |
| Pb5^i^-Pb5 | 3.5408(7) |  |  |
| Pb5-Pb2^i^ | 3.1834(5) |  |  |
| Pb2^i^-Pb4^i^ | 3.8441(6) |  |  |
| Pb4^i^-Pb3^i^ | 3.0823(5) |  |  |
| Pb5^i^-Pb3^i^ | 3.0738(5) |  |  |
| Pb5-Pb3^i^ | 3.0903(5) |  |  |
| Pb2^i^-Pb3^i^ | 3.0973(5) |  |  |
| Pb1-Pb3 | 4.7589(5) |  |  |
| Pb2-Pb4 | 3.8441(6) |  |  |

**Table S12**: Shortest possible hydrogen bonds in **3**, distances in Å.

| **Atoms** | **N-H distances** | **H∙∙∙Pb distances** | **N∙∙∙Pb distances** | **N-H∙∙∙Pb angles** |
| --- | --- | --- | --- | --- |
| N1-H1d∙∙∙Pb1^i^ | 0.91 | 3.06 | 3.915(7) | 157.8° |
| N5-H5c∙∙∙Pb5^iii^ | 0.91 | 3.06 | 3.956(7) | 167.3° |
| N6-H6d∙∙∙Pb3^ii^ | 0.91 | 3.07 | 3.897(7) | 151.5° |
| N1-H1c∙∙∙Pb2^ii^ | 0.91 | 3.15 | 3.894(7) | 140.8° |
| N4-H4d∙∙∙Pb3^ii^ | 0.91 | 3.17 | 3.987(6) | 150.7° |

**Table S13**: Selected interatomic distances in **4**.

| Atoms | Distance [Å] | Atoms | Distance [Å] |
| --- | --- | --- | --- |
| Ge1-Ge1^i^ | 2.503(4) | Ge10-Ge10^i^ | 2.525(6) |
| Ge1-Ge2 | 2.537(2) | Ge10-Ge11 | 2.455(3) |
| Ge2-Ge3 | 2.607(2) | Ge11-Ge12 | 2.641(3) |
| Ge3-Ge4 | 2.604(2) | Ge12-Ge13 | 2.665(3) |
| Ge4-Ge1 | 2.512(2) | Ge13-Ge10 | 2.568(5) |
| Ge1-Ge5 | 2.601(2) | Ge10-Ge14 | 2.633(4) |
| Ge1-Ge6 | 2.581(4) | Ge10-Ge15 | 2.728(8) |
| Ge2-Ge6 | 2.645(5) | Ge11-Ge15 | 2.778(8) |
| Ge2-Ge7 | 2.563(4) | Ge11-Ge16 | 2.546(5) |
| Ge3-Ge7 | 2.625(4) | Ge12-Ge16 | 2.610(5) |
| Ge3-Ge8 | 2.600(2) | Ge12-Ge17 | 2.608(3) |
| Ge4-Ge8 | 2.583(2) | Ge13-Ge17 | 2.536(3) |
| Ge4-Ge5 | 2.704(3) | Ge13-Ge14 | 2.644(4) |
| Ge5-Ge6 | 2.880(4) | Ge14-Ge15 | 2.825(3) |
| Ge6-Ge7 | 2.721(6) | Ge15-Ge16 | 2.792(9) |
| Ge7-Ge8 | 2.968(4) | Ge16-Ge17 | 2.918(4) |
| Ge8-Ge5 | 2.721(2) | Ge17-Ge14 | 2.780(3) |
| Ge5-Ge9 | 2.553(3) | Ge14-Ge18 | 2.578(8) |
| Ge6-Ge9 | 2.582(5) | Ge15-Ge18 | 2.531(11) |
| Ge7-Ge9 | 2.593(4) | Ge16-Ge18 | 2.570(7) |
| Ge8-Ge9 | 2.575(3) | Ge17-Ge18 | 2.575(6) |
| Ge1-Ge3 | 3.580(2) | Ge10-Ge12 | 3.427(4) |
| Ge2-Ge4 | 3.667(3) | Ge11-Ge13 | 3.860(4) |
|  |  |  |  |
| Li1-N1 | 2.018(11) | Li2-N2 | 2.067(10) |
| Li1-N3 | 2.083(11) | Li2-N4 | 2.089(10) |
| Li1-N5^i^ | 2.088(12) | Li2-N6 | 2.095(11) |
| Li1-N13 | 2.174(19) | Li2-N11 | 2.045(10) |
| Li1-N14 | 1.947(18) | Li3-N7 | 2.101(12) |
|  |  | Li3-N8 | 2.060(12) |
|  |  | Li3-N9 | 2.052(12) |
|  |  | Li3-N12 | 2.061(12) |

**Table S14**: Shortest possible hydrogen bonds in **4**, distances in Å.

| **Atoms** | **N-H distances** | **H∙∙∙Ge distances** | **N∙∙∙Ge distances** | **N-H∙∙∙Ge angles** | |
| --- | --- | --- | --- | --- | --- |
| N4-H4d∙∙∙Ge4^ii^ | 0.91 | 2.68 | 3.563(2) | 164.8° | |
| N1-H1d∙∙∙Ge2^i^ | 0.91 | 2.70 | 3.576(2) | 162.5° | |
| N1-H1c∙∙∙Ge9 | 0.91 | 2.76 | 3.670(2) | 175.4° | |
| N2-H2c∙∙∙Ge5^iii^ | 0.91 | 2.80 | 3.686(2) | 164.5° | |
| N5-H5c∙∙∙Ge8^iv^ | 0.91 | 2.80 | 3.695(2) | 167.0° | |
| N5-H5d∙∙∙Ge3 | 0.91 | 2.85 | 3.666(2) | 149.3° |  |
| N3-H3c∙∙∙Ge7 | 0.91 | 2.88 | 3.635(2) | 141.1° |  |
| N2-H2d∙∙∙Ge2^i^ | 0.91 | 2.92 | 3.687(3) | 142.6° |  |

**Table S15**: Selected interatomic distances in **5**.

| Atoms | Distance [Å] | Atoms | Distance [Å] |
| --- | --- | --- | --- |
| Ge1-Ge1^i^ | 2.5106(14) | Ge4-K | 3.3805(18) |
| Ge1-Ge2 | 2.5430(10) | Ge2-K | 3.4547(18) |
| Ge2-Ge3 | 2.6234(10) | Ge6-K | 3.4769(18) |
| Ge3-Ge4 | 2.6073(10) | Ge8-K | 3.4785(18) |
| Ge4-Ge1 | 2.5582(10) | Ge4-K | 3.5705(18) |
| Ge1-Ge5 | 2.6232(10) | Ge5-K | 3.6268(19) |
| Ge1-Ge6 | 2.6189(10) | Ge1-K | 3.7864(18) |
| Ge2-Ge6 | 2.6907(10) | Ge1-K | 3.8635(18) |
| Ge2-Ge7 | 2.5802(10) | Ge5-K | 3.9156(19) |
| Ge3-Ge7 | 2.6156(10) |  |  |
| Ge3-Ge8 | 2.5908(10) | Li1-N1 | 2.122(13) |
| Ge4-Ge8 | 2.6052(10) | Li1-N3 | 2.082(13) |
| Ge4-Ge5 | 2.6858(10) | Li1-N5 | 2.054(13) |
| Ge5-Ge6 | 2.8335(10) | Li1-N7 | 2.046(12) |
| Ge6-Ge7 | 2.7322(10) | Li2-N2 | 2.096(12) |
| Ge7-Ge8 | 2.8527(10) | Li2-N4 | 2.111(13) |
| Ge8-Ge5 | 2.7747(10) | Li2-N6 | 2.109(13) |
| Ge5-Ge9 | 2.5932(10) | Li2-N8 | 2.099(12) |
| Ge6-Ge9 | 2.5817(10) |  |  |
| Ge7-Ge9 | 2.5906(10) |  |  |
| Ge8-Ge9 | 2.5713(10) |  |  |
| Ge1-Ge3 | 3.5040(10) |  |  |
| Ge2-Ge4 | 3.7942(10) |  |  |

**Table S16**: Shortest possible hydrogen bonds in **5**, distances in Å.

| **Atoms** | **N-H distances** | **H∙∙∙Ge distances** | **N∙∙∙Ge distances** | **N-H∙∙∙Ge angles** |
| --- | --- | --- | --- | --- |
| N5-H5d∙∙∙Ge3 | 0.91 | 2.86 | 3.673(5) | 149.2° |
| N1-H1d∙∙∙Ge3 | 0.91 | 2.90 | 3.719(6) | 150.0° |
| N5-H5c∙∙∙Ge9^ii^ | 0.91 | 2.91 | 3.824(5) | 177.5° |
| N3-H3d∙∙∙Ge7^i^ | 0.91 | 2.93 | 3.520(6) | 124.4° |
| N7-H7d∙∙∙Ge7 | 0.91 | 2.99 | 3.777(6) | 145.2° |
| N2-H2d∙∙∙Ge9^iii^ | 0.91 | 3.01 | 3.915(6) | 172.2° |
| N8-H8d∙∙∙Ge7 | 0.91 | 3.02 | 3.930(6) | 178.9° |
| N2-H2c∙∙∙Ge2^i^ | 0.91 | 3.06 | 3.898(6) | 154.1° |

# References

[1] H. G. Von Schnering, M. Baitinger, U. Bolle, W. Carrillo-Cabrera, J. Curda, Y. Grin, F. Heinemann, J. Llanos, K. Peters, A. Schmeding, M. Somer, *Z. Anorg. Allg. Chem.* **1997**, *623*, 1037-1039.

[2] S. Frischhut, M. M. Bentlohner, T. F. Fässler, *Eur. J. Org. Chem.* **2019**, *2019*, 3101-3104.

[3] S. Ponou, T. F. Fässler, *Z. Anorg. Allg. Chem.* **2007**, *633*, 393-397.

[4] I. F. Hewaidy, E. Busmann, W. Klemm, *Z. Anorg. Allg. Chem.* **1964**, *328*, 283-293.

[5] Y. Grin, M. Baitinger, R. Kniep, H. G. v. Schnering, *Z. Kristallogr. - N. Cryst. Struct.* **1999**, *214*, 453-454.

[6] M. Somer, U. Aydemir, M. Baitinger, H. G. von Schnering, *Z. Anorg. Allg. Chem.* **2006**, *632*, 1281-1286.

[7] M. Somer, W. Carrillo-Cabrera, E. M. Peters, K. Peters, H. G. v. Schnering, *Z. Anorg. Allg. Chem.* **1998**, *624*, 1915-1921.

[8] W. Carrillo-Cabrera, U. Aydemir, M. Somer, A. Kircali, T. F. Fässler, S. D. Hoffmann, *Z. Anorg. Allg. Chem.* **2007**, *633*, 1575-1580.

[9] J. D. Corbett, P. A. Edwards, *J. Am. Chem. Soc.* **1977**, *99*, 3313-3317.

[10] R. Hauptmann, T. F. Fässler, *Z. Kristallogr. - N. Cryst. Struct.* **2003**, *218*, 458-460.

[11] A. Ugrinov, S. C. Sevov, *Appl. Organomet. Chem.* **2003**, *17*, 373-376.

[12] W. Klein, H. He, T. F. Fässler, *Acta Crystallogr., Sect. E* **2017**, *73*, 147-151.

[13] R. Hauptmann, R. Hoffmann, T. F. Fässler, *Z. Anorg. Allg. Chem.* **2001**, *627*, 2220-2224.

[14] T. F. Fässler, R. Hoffmann, *J. Chem. Soc., Dalton Trans.* **1999**, 3339-3340.

[15] L. Yong, S. D. Hoffmann, T. F. Fässler, *Inorg. Chim. Acta* **2006**, *359*, 4774-4778.

[16] J. Campbell, D. A. Dixon, H. P. A. Mercier, G. J. Schrobilgen, *Inorg. Chem.* **1995**, *34*, 5798-5809.

[17] K. Mayer, W. Klein, S. Geier, T. F. Fässler, *Z. Anorg. Allg. Chem.* **2021**, *647*, 377-384.

[18] A. Ugrinov, S. C. Sevov, *J. Am. Chem. Soc.* **2003**, *125*, 14059-14064.

[19] R. Hauptmann, T. F. Fässler, *Z. Kristallogr. - N. Cryst. Struct.* **2003**, *218*, 461-463.

[20] A. Nienhaus, S. D. Hoffmann, T. F. Fässler, *Z. Anorg. Allg. Chem.* **2006**, *632*, 1752-1758.

[21] C. Wallach, W. Klein, T. F. Fässler, *Z. Anorg. Allg. Chem.* **2022**, *648*, e202200065.
